# RAD genotyping reveals fine-scale population structure and provides evidence for adaptive divergence in a commercially important fish from the northwestern Pacific Ocean

**DOI:** 10.7717/peerj.7242

**Published:** 2019-07-03

**Authors:** Bai-Dong Zhang, Dong-Xiu Xue, Yu-Long Li, Jin-Xian Liu

**Affiliations:** 1 CAS Key Laboratory of Marine Ecology and Environmental Sciences, Institute of Oceanology, Chinese Academy of Sciences, Qingdao, China; 2 Laboratory for Marine Ecology and Environmental Science, Qingdao National Laboratory for Marine Science and Technology, Qingdao, China; 3 Center for Ocean Mega-Science, Chinese Academy of Sciences, Qingdao, China

**Keywords:** Population genomics, Single-nucleotide polymorphisms, RAD-seq, Local adaptation, *Larimichthys polyactis*

## Abstract

Exploring factors shaping genetic structure of marine fish is challenging due to fewer barriers to gene flow in the ocean. However, genome-wide sequence data can greatly enhance our ability to delineate previously unidentified population structure as well as potential adaptive divergence. The small yellow croaker (*Larimichthys polyactis*) is a commercially important fish species with high gene flow and its overwintering populations experience heterogeneous environment, suggesting possible population differentiation and adaptive divergence. To delineate patterns of population structure as well as test for signatures of local adaptation, a total of 68,666 quality filtered SNP markers were identified for 80 individuals from four overwintering populations by using restriction site-associated DNA sequencing (RAD-seq). Significant genetic differentiation among overwintering populations from the Central Yellow Sea, the South Yellow Sea and the North East China Sea were detected (Pair-wise *F*_ST_: 0.00036–0.00390), which were consistent with population division of overwintering groups inferred from traditional ecological approaches. In addition, a total of 126 unique SNPs were detected to be significantly associated with environmental parameters (temperature, salinity and turbidity). These candidate SNPs were involved in multiple pathways such as energy metabolism and phagocytosis, suggesting they may play key roles in growth and innate immunity. Our results suggested the existence of hitherto unrecognized cryptic population structure and local adaptation in this high gene flow marine fish and thus gain new insights into the design of management strategies.

## Introduction

Knowledge on the ecological and evolutionary processes influencing biodiversity and dispersal of marine organisms is fundamental to the understanding of their genetic distribution patterns and provides an explicit framework for conservation and management of resource species (Nielsen et al., 2009a; Conover et al., 2006). Population structure in marine fishes was believed to be very limited, which might be attributed to fewer barriers to gene flow than on land as well as large population sizes, resulting in relatively high levels of connectivity among populations (Shanks, Grantham & Carr, 2003; Conover et al., 2006; Cano et al., 2008). Therefore, adaptive divergence is considered to be limited or absent in marine fishes, because natural selection would have to override genetic drift as well as the constant gene flow among populations. However, on the other hand, large population sizes reduce the effect of genetic drift and increase the likelihood of preserving favorable alleles due to local selective pressures (Nielsen et al., 2009b). For the last decades, genetic data from putatively neutral markers such as microsatellites, mitochondrial DNA and amplified fragment length polymorphisms (AFLPs) have been extensively used in population genetic studies of marine fishes, however, these applications have shown the main limitation in reflecting effects of selection or environmental influences (Billington & Hebert, 1990; Jarne & Lagoda, 1996; Campbell, Duchesne & Bernatchez, 2003). Genome scan approaches, such as restriction site-associated DNA sequencing (RAD-seq), present a framework for identifications of genome-wide SNP markers in natural populations and thus elucidating directional selections by identifying outlier loci with elevated levels of genetic differentiation (Storz, 2005; Miller et al., 2007; Allendorf, Hohenlohe & Luikart, 2010; Etter et al., 2011). Although disentangling adaptive genetic variation in marine fish species has become a renewed challenge in evolutionary biology, there is increasing evidence that adaptive divergence and cryptic population structure may indeed be prevalent in most marine fishes despite seemingly high levels of gene flow. Guo, Li & Merilä (2016) elucidated genome-wide levels of divergence of Atlantic herring (*Clupea harengus*) in the Baltic Sea and detected genomic regions associated with temperature and salinity related natural selection. Le Moan, Gagnaire & Bonhomme (2016) documented the extent of genetic differentiation between coastal and marine ecotypes of the European anchovy (*Engraulis encrasicolus*) from Atlantic and Mediterranean, which provides comprehensive insights into the understanding of fine-scale genetic structuring in European anchovy and thus improves stock management and conservation actions. Additionally, Babin et al. (2017) evaluated the effects of spatially varying selection in shaping genetic variation across the genome of American Eel (*Anguilla rostrata*) and outlier loci detected were found to be significantly associated with environmental variables such as latitude, longitude as well as temperature.

The small yellow croaker (*Larimichthys polyactis*) is a benthopelagic fish of the family Sciaenidae, widely distributed in northwestern Pacific Ocean, including the Bohai Sea, the Yellow Sea and the East China Sea (FishBase 2014). *L. polyactis* is a multiple spawner with seasonal asynchronous migrations (Xu & Chen, 2009). Estuaries or coastal waters are major spawning and nursery areas for *L. polyactis* and the overwintering grounds are located in offshore area (Chen, Xu & Chen, 2010). *L. polyactis* migrate from overwintering grounds to spawning grounds in early April and begin spawning in the coastal waters from late April of the East China Sea to June of the Yellow Sea and the Bohai Sea with a gradually spatial and temporal change (Liu, 1962; Xu & Chen, 2009). After spawning, fish groups continuously move to nearby coastal waters for feeding and after October, they migrate eastward back to overwintering grounds offshore (Liu, 1962; Xu & Chen, 2009). The number of *L. polyactis* had sharply declined in China since the 1970s due to factors such as overfishing, marine pollution as well as changes of water masses and currents, and after the declaration of China’s Summer Fishing Moratorium in 1990s, the resources of *L. polyactis* had gradually recovered. (Cheng et al., 2004; Jin, 2004; Ding et al., 2007; Li, Lin & Cheng, 2009). Nevertheless, overharvesting pressure has adversely influenced the natural resources of this species and distinct trends of biological characteristics changes have been observed, such as earlier maturation, lower age composition as well as miniaturization (Jin, 1996; Lin & Cheng, 2004). In the last decades, population division of *L. polyactis* in overwintering grounds has been one of research focuses in the field of fishery resources and ecology, however, traditional studies built upon catch data, morphology, anatomy and migration behavior have generated inconsistent results (Liu, 1962; Wang, Ma & You, 1965; Xu & Chen, 2009). Hence, a better understanding of population structure of *L. polyactis* in overwintering grounds is urgently needed, which should provide new insights into the design of management strategies for this invaluable fishery resource.

**Figure 1:**
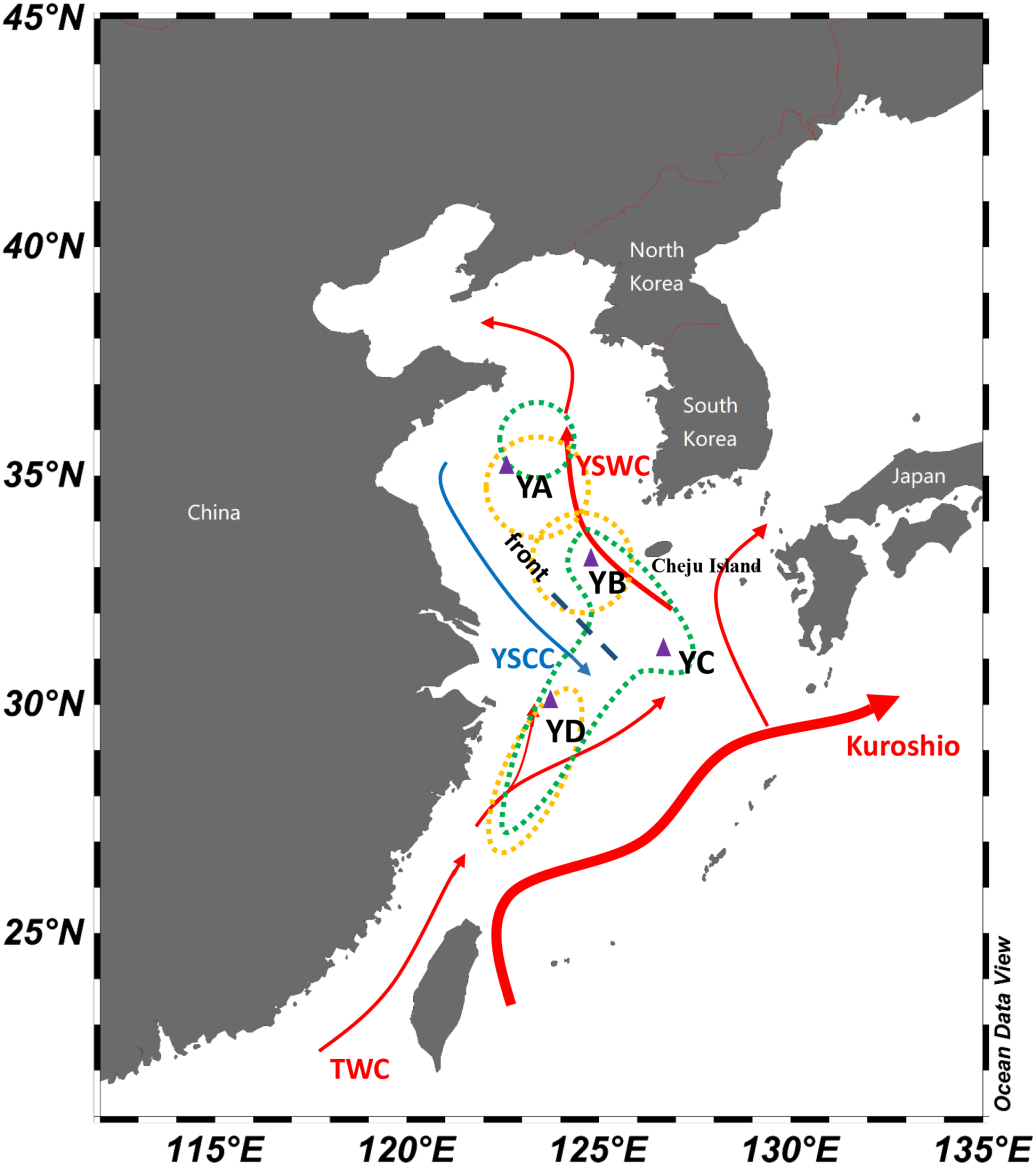
Schematic map of winter currents in the Yellow Sea and East China Sea. The red arrows denote the warm currents and the blue arrows denote the cold currents. The dashed lines represent the hydrological front in the central Yellow Sea. The triangles represent sampling locations in overwintering grounds of *L. polyactis*. The yellow dots denote population division of *L. polyactis* in overwintering grounds based on the studies of Wang, Ma & You (1965) and Lin et al. (1964) and the green dots denote population division of *L. polyactis* in overwintering grounds according to Xu & Chen (2009) and Xu & Chen (2011) studies (Schlitzer, 2019).

*L. polyactis* represents one of the most economically important fishery resources in East Asian countries and has become the subject of numerous population genetic studies. However, lack of strict natal homing as well as admixture of isolated overwintering groups make them an anomaly among other migratory fishes, which has added to mystery surrounding the species and thus made it more challenging to uncover possible cryptic structure and adaptive divergence in *L. polyactis*. Previous fishery ecological studies suggested three overwintering groups including the Bohai Sea and Yellow Sea group, the Southern Yellow Sea group, and the East China Sea group (Wang, Ma & You, 1965) (Fig. 1). However, Xu & Chen (2009) and Xu & Chen (2011) demonstrated that there were two instead of three overwintering groups of *L. polyactis* in the China Sea, including the Northern Yellow Sea and Bohai Sea group and the Southern Yellow Sea and East China Sea group (Fig. 1). In previous population genetic studies, limited genetic data from narrow regions of the genome were used to delineate population structure and discrepancies in these results of genetic structure of *L. polyactis* have to some extent hindered the effectiveness of fisheries management and conservation (Meng et al., 2003; Lin et al., 2009; Xiao et al., 2009; Kim et al., 2010; Wu et al., 2012; Kim et al., 2012; Li et al., 2013; Zhang et al., 2014).

Under the influences of oceanographic factors such as ocean currents as well as monsoons, the offshore areas for overwintering grounds during winter seasons may have a relatively stable and steep pattern of environmental parameters on small spatial scales, providing chances for local adaptation (Yu et al., 2010). The 31 year (from 1976 to 2007) average winter observations from the SOA (State Oceanic Administration) routine surveys revealed clearly the presence of warmer and saltier water column on the western part of the Yellow Sea Trough in winter and this pattern is consistent with the path of the Yellow Sea Warm Current (YSWC) (Lin et al., 2011). Moreover, the Yellow Sea Cold Water Mass (YSCWM), the most dominant water mass, forms in deeper part of the Yellow Sea by vigorous vertical mixing under strong wind, and the water column becomes almost vertically uniform in winter (Oh et al., 2013). In seasonal cycles, hydrographic characteristics of China Seas are mainly influenced by monsoons as well as ocean currents, where environmental conditions such as temperature, salinity and turbidity show distinct environmental transitions. Therefore, environmental heterogeneity in the offshore area may influence the genetic variation of *L. polyactis* at different spatial scales. However, earlier studies based on microsatellite markers only detected several loci under directional selection in *L. polyactis* (Wang et al., 2013; Liu et al., 2016). The advent of high throughput next-generation sequencing, such as restriction site-associated DNA Sequencing (RAD-seq) (Miller et al., 2007; Davey & Blaxter, 2010), facilitates the identification of signatures of local adaptation in marine fish through genome-wide scans. This happens because shallow genetic structure of high gene flow marine fish exhibits lower neutral background noises of genetic divergence and selective loci would tend to be distinguished as outliers (Allendorf, Hohenlohe & Luikart, 2010; Pérez-Figueroa et al., 2010). In these circumstances, population-genomic approach using genome-wide markers may provide a more complete picture of genetic variation in overwintering populations of *L. polyactis* and gain new insights into the degree and scale of genetic differentiation as well as adaptive divergence (Luikart et al., 2003).

In this study, we applied a population-genomic approach using RAD-seq to genotype 80 individuals of *L. polyactis* collected from four geographical overwintering populations. According to previous fishery dependent studies, the above four locations were chosen to represent the main distribution areas of overwintering aggregations of this species (Liu, 1962; Wang, Ma & You, 1965; Xu & Chen, 2009). The aim of this study was to identify a set of genome-wide SNP markers to uncover possible cryptic structure in this species which previous studies may have failed to discern. Furthermore, the association with environmental parameters of overwintering grounds (salinity, temperature and turbidity) was also tested to identify signatures of local adaptation. Together, the results should advance our understanding of population structure and adaptive divergence of *L. polyactis*, which is crucial for identifying and designating management units and thus ensure its long-term sustainability and resilience in relation to environmental and climate changes for this fish (Hilborn et al., 2003; Schindler et al., 2010).

## Materials & Methods

### Sample collection

This study focuses on overwintering populations of *L. polyactis* from the Yellow Sea and East China Sea. According to previous fishery resources and ecology studies of *L. polyactis* (Wang, Ma & You, 1965; Xu & Chen, 2009), fish specimens were collected from four main overwintering aggregations including the Central Yellow Sea (YA), the South Yellow Sea (YB), South of Cheju island (YC), and the North East China Sea (YD) in November 2016 (Table 1; Fig. 1). All individuals were taken from “The First Open Cruise of the Yellow Sea and East China Sea Survey (2016)” conducted by Qingdao National Laboratory for Marine Science and Technology during overwintering seasons of *L. polyactis*, ensuring the collected samples were representatives of the overwintering populations. All individuals were collected by trawling net. Individual muscle tissue were preserved in 95% ethanol and genomic DNA was extracted using standard phenol–chloroform extraction protocol. In total, 80 individuals (20 individuals from each population) were used for RAD genotyping.

**Table 1:** Sampling locations, year, latitudinal range, sample size, and environmental variables for each collection of *L. polyactis* in this study.

Location	Code	Sample size	Sample time	Coordinates		Average temperature (deg C)	Average salinity (PSU)	Average turbidity (NTU)
Central Yellow Sea	YA	20	2016-11-13	35°00′40″N	122°33′32″E	12.30	32.07	7.81
South Yellow Sea	YB	20	2016-11-17	32°59′49″N	124°08′06″E	15.68	32.10	32.83
South of Cheju Island	YC	20	2016-11-19	31°02′30″N	126°16′30″E	20.76	33.88	28.14
North East China Sea	YD	20	2016-11-25	29°59′26″N	123°26′50″E	21.49	33.75	5.36

### RAD library construction and sequencing

To produce pure, high molecular weight, RNA-free DNA, genomic DNA was treated with RNase A to remove RNA contamination. RAD libraries were prepared following the guidelines in the protocol described in Baird et al. (2008). For each individual, about 500 ng DNA was digested using *Eco* RI. RAD libraries were constructed using pools of 10–12 individually indexed individuals and DNA fragment of 200–400 bp was isolated and purified. The libraries were enriched by high-fidelity PCR prior to paired-end sequencing from the two lanes of Illumina HiSeq4000 at Novogene in Beijing, resulting in read lengths of 150 bp. Sequence reads from the Illumina runs were sorted according to index sequences (six nucleotides in length and each differed by at least two bases) for individual tracking. To avoid reads with artificial bias, the process_radtags program (Catchen et al., 2013) and Cutadapt v1.16 (Martin, 2011) were used to remove low-quality read pair with the following criteria: (1) Remove read pairs with adapter contamination; (2) Remove read pairs with ≥ 5% unidentified nucleotides; (3) Read pairs having average phred quality <20 within a window size of 15 bp were removed; (4) Discard putative duplicated read pairs generated by PCR amplification; (5) Remove read pairs without the presence of the partial *Eco* RI motif (AATTC). Final read length of the first read (containing the restriction-site) was trimmed to 125 nucleotides to avoid the potential sequencing errors at the tails of the sequences (Pujolar et al., 2013).

### Assembly and SNP identification

Stacks v2.0 (Catchen et al., 2013) was applied to process the data. By using ustacks, single-end loci in each individual was built by grouping unique stacks of possible alleles. The minimum depth of coverage to create a stack was set at a conservative value of 4 (m parameter in ustacks), and the maximum number of pairwise differences between stacks was set 6 (M parameter in ustacks) according to the study of Ilut, Nydam & Hare (2014) by using RADassembler (Li et al., 2018). In addition, gapped alignments (gapped parameter in ustacks) were preformed between stacks and the other parameters were set to default values. Twenty two individuals (five individuals from each of the two populations: YA and YB; six individuals from each of the two populations: YC and YD) of *L. polyactis* were used to build the catalog using cstacks program with default parameters except that –n was set to 5 (Ilut, Nydam & Hare, 2014; Li et al., 2018). First reads of each individual were matched back to the catalog using sstacks with default parameters. In addition, the gstacks program is executed with default parameters to assemble contigs, call variant sites using all matched reads for each RAD locus of the catalog and genotypes in each individual. After assembly and genotyping, the data was further filtered to ensure maximal quality by using VCFtools (Danecek et al., 2011). In this study, only single nucleotide substitutions were focused on and other complex events such as indels and multinucleotide polymorphisms were ingnored. For instance, loci with more than two alleles were discarded to avoid potential sequencing errors. In addition, SNPs with minor allele frequency (Global MAF) >0.05 across populations were kept to reduce false SNP identification (Corander et al., 2013). Further, the following quality controls were also included for SNP calling: (1) Coverage depth ≥ 8 and ≤ 1,000; (2) genotype quality score ≥ 15; (3) SNPs with a minimum genotyping rate of 80% within each population was kept. In order to avoid potential homeologs, markers showing global *H*_obs_ > 0.50 and —*F*_IS_— ≥ 0.3 were excluded. SNPs that passed the quality control threshold were retained in the VCF output file for the following analysis. The resulting filtered VCF file was converted into the corresponding file formats for the subsequent analyses by using PGDSPIDER v.2.0.5.0 (Lischer & Excoffier, 2012).

### Population genetic analyses

As most loci located at the same genome neighborhood might show physical linkage, only one SNP was randomly retained for each RAD locus to avoid linkage disequilibrium in the following analyses. Discriminant analysis of principal components (DAPC, Jombart, Devillard & Balloux, 2010) was applied to conduct population structure analysis using Adegenet (Jombart, 2008) for R (R Development Core Team, 2015) and the number of principal components was determined based on optim-alpha-score indication. We performed a DAPC with prior information of sampling locations (*K* = 4) as the DAPC without prior did not reveal any genetic structure (number of genetic clusters inferred was *K* = 1). For comparison, principal component analysis (PCA) colored by sampling population was also conducted to illustrate the results of population structure. Moreover, we assessed the distribution of genetic variation across samples by using a Bayesian model-based clustering program ADMIXTURE v1.23 (Alexander, Novembre & Lange, 2009). We varied the number of clusters, *K* (from 1 to 5 with ten replicates for each value), until we had the optimal number of clusters by plotting the natural probability of four estimators (MEDMEDK, MEDMEAK, MAXMEDK and MAXMEAK) and the *K* value was determined where the estimators plateaued as described in Puechmaille (2016). We used STRUCTURESELECTOR (Li & Liu, 2018) to select the most likely *K* for individual estimator and graphical representations of the results were generated by integrating the CLUMPAK program (Kopelman et al., 2015). VCFtools (Danecek et al., 2011) was applied to reformat input files into PLINK format files (MAP/PED) for ADMIXTURE. Furthermore, Average pairwise *F*_ST_ was calculated between each pair of sampling populations using ARLEQUIN v3.5.1.3 (Excoffier & Lischer, 2010) and significance was determined using 10,000 permutations.

### Detection of local adaptation associated with environmental variables

To detect loci involved in local adaptation, a Bayesian approach as implemented in Bayenv 2.0 was applied to test for association between allele frequencies and environmental variables (Coop et al., 2010; Günther & Coop, 2013). To avoid linkage disequilibrium, a total of 21,620 loci (one random SNP per locus) were used to generate the covariance matrix. The Bayesian approach allows for the effects of population structure and a mean covariance matrix was calculated from allelic data (using the final run of the MCMC after 2 × 10^5^ interactions) to control evolutionary history in the calculation of *X*^*T*^*X* for individual SNP (using 2 × 10^5^ iterations of the MCMC) (Coop et al., 2010; Günther & Coop, 2013). Three potential explanatory environmental variables including temperature, salinity and turbidity were tested for association with allele frequencies. Environmental parameter data of the four overwintering-population sites were acquired from the conductivity-temperature-depth (CTD) measurements by a Sea-Bird SBE19plus CTD from “The First Open Cruise of the Yellow Sea and East China Sea Survey” by Qingdao National Laboratory for Marine Science and Technology in November 2016 along with sample collection. Furthermore, these three environmental parameters acquired from previous research survey of “The Open Cruise of Chinese Offshore Oceanography Research” by Institute of Oceanology, Chinese Academy of Sciences in November 2012 were also included in the present study. To reduce temporal variation in marine environment of the overwintering grounds, environmental data of the four sampling locations were averaged over 2012 and 2016 (Table 1). Observations in both of the two years were made during November in the same overwintering regions and at each station, temperature, salinity and turbidity were measured with a high vertical resolution of less than 0.1 m. Considering the depth distribution of *L. polyactis* in overwintering grounds, average environmental variable within depth of 40–80 m was used to test for association with genetic variation. We calculated Pearson’s *r* correlation and associated significance levels between environmental variables using the rcorr function in the R base package (R Development Core Team, 2015). The environmental data was standardized by subtracting the mean and dividing through by the standard deviation of the variable. Three independent runs (differing in the random seed) were run to ensure the results generated were not sensitive to stochastic errors.

### Genome alignment and annotation of RAD loci

RAD loci associated with the ecological variables were further characterized by whether they were located within or linked to a gene by aligning the RAD sequences to the previously published genome of *Larimichthys crocea* (GenBank assembly accession: GCA_000972845.1), a sister species of *L. polyactis* (Ao et al., 2015), using Standard Nucleotide BLAST (*E*-value ≤ 10^−6^) in National Center for Biotechnology Information Search database (NCBI). The scaffolds of *L. crocea* have been assembled and annotated including both putative and known coding regions, we aim to evaluate the genomic locations of RAD loci and provide annotations for coding regions harboring RAD loci as well as coding sequences within 5 kb of the mapped RAD loci (Nichols, Kozfkay & Narum, 2016). The NCBI Genome Data Viewer (GDV, https://www.ncbi.nlm.nih.gov/genome/gdv/) was used to visualize coding-sequence (CDS) associated RAD loci in the *L. crocea* genome. In addition, Kyoto Encyclopedia of Genes and Genomes (KEGG) pathway approach implemented in the Database for Annotation, Visualization and Integrated Discovery (DAVID) web-server v6.8 (https://david.ncifcrf.gov/) was further applied for functional annotation and classification of the candidate genes (Huang, Sherman & Lempicki, 2009a; Huang, Sherman & Lempicki, 2009b).

## Results

### RAD sequencing and data filtering

The average number of quality filtered reads per individual was approximately 10.98 million reads (range from 4,401,358 to 39,202,588; Table S1). The assembly of reads from all individuals for each RAD locus produced a total of approximately 336,858 contigs with an average length of 402.3 bp. Quality filtering resulted in 68,666 SNPs and after keeping only one SNP per locus, a total of 21, 620 SNPs were finally retained for the following analyses. Summary statistics for counts of putative SNP loci and final counts of candidate SNPs after each filtering step are available in Table 2.

**Table 2:** Summary statistics for counts of putative SNP loci and final counts of candidate SNPs after different filtering steps.

Filtering step	SNP counts
Biallelic variants	5,127,919
Global minor allele frequency (MAF > 0.05)	2,764,201
8 ≤ Coverage depth ≤ 1,000 Genotype quality score ≥ 15 Coverage ratio of samples for each pop ≥ 80%	78,871
Inbreeding coefficient (−0.3 < Fis < 0.3) Heterozygosity (H ≤ 0.5)	68,666
Keep one SNP of each contig	21,620

### Population genetic analyses

**Figure 2:**
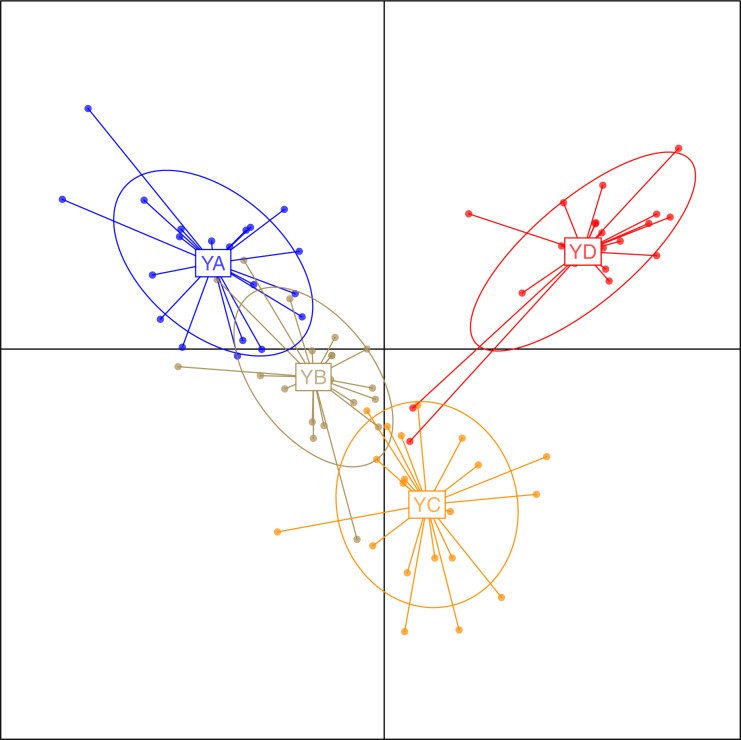
Discriminant analysis of principal components (DAPC) plots for 21,620 SNPs in four overwintering populations of *L. polyactis*. Ellipse centers of each cloud of points represent each sampling location.

**Figure 3:**
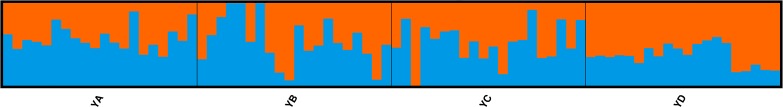
ADMIXTURE clustering results for *K* = 2 based on the data set of 21,620 SNPs.

To investigate the extent of population structuring in overwintering populations of *L. polyactis*, DAPC was performed with prior information of sampling locations (*K* = 4) as the DAPC without prior did not reveal any genetic structure (number of genetic clusters inferred was *K* = 1, Fig. S1). Results of the DAPC analysis using the first two principle axes indicated two dominant groups representing the East China Sea group (YD) and the Yellow Sea group (YA, YB, YC). In addition, DAPC analysis also demonstrated that no overlap was found between YA and YC in the Yellow Sea group and YB appeared as an intermediate population between them (Fig. 2). Similar results were also observed using a PCA analysis of the same dataset (Fig. S2). In ADMIXTURE, the optimum value of K identified by cross-validation error was observed to be *K* = 1 (Fig. S3). However, the most likely number of genetic clusters was determined to be *K* = 2 based on new supervised estimators of MEDMEDK, MEDMEAK, MAXMEDK and MAXMEAK (Fig. S4). A similar pattern was evident in the analyses of ADMIXTURE (Fig. 3) and all four populations were split into two groups: the East China Sea group (YD) and an admixed Yellow Sea group consisting of YA, YB, YC, which was consistent with the results revealed using DAPC. Additionally, it is worth to note that the optimum value of MAXMEAK was observed to be 3 (Fig. S4), indicating the potential existence of three groups among these populations. Similarly, the pairwise *F*_ST_ between YD and the other populations ranged from 0.00218 to 0.00379 and were all statistically significant (*P* <0.05, Table 3). No differentiation was observed between YB and YC. In addition, significant levels of genetic differentiation were also obtained for both population comparisons of YA-YB and YA-YC (*P* < 0.05, Table 3). In contrast to the group membership inferred from DAPC and ADMIXTURE, results of population pairwise *F*_ST_ revealed that the Yellow Sea group consisting of YA, YB, YC was further split into two groups of the Central Yellow Sea group (YA) and the South Yellow Sea group (YB, YC), although the differentiation between them was to some extent shallow. Taken together, these results indicated three genetically differentiated groups in the overwintering grounds of *L. polyactis*, including the Central Yellow Sea group (YA), the South Yellow Sea group (YB, YC) and the North East China Sea group (YD).

### Detection of loci associated with environmental variables

In the present study, no significant association between environmental variables was observed based on Pearson’s correlation coefficient. The BAYENV analysis based on the data set of 21,620 SNPs revealed that some SNPs across the genome were associated with the environmental parameters (temperature, salinity and turbidity). On the basis of criterion of log10 Bayes factor (BF) greater than 3, we found that 52 SNPs were correlated with variation in temperature, 46 SNPs were correlated with salinity variation and 40 SNPs were found to be correlated with turbidity, respectively (Table S2). Of all the SNPs significantly associated with the three environmental parameters, 12 SNPs were observed to be associated with both temperature and salinity. Overall, a total of 126 unique RAD loci harboring the SNPs associated with environmental variation were used for subsequent gene annotations.

**Table 3:** Pairwise *F*_*ST*_ among four overwintering populations of *L. polyactis* based on the dataset of 21,620 SNPs.

	YA	YB	YC	YD
YA	–	–	–	–
YB	0.00046^*^	–	–	–
YC	0.00036^*^	0.00000	–	–
YD	0.00379^*^	0.00218^*^	0.00229^*^	–

**Notes.**

* Values of *F*_*ST*_ that significantly differed from zero (*p* < 0.05 after Bonferroni correction).

### Genome alignment and annotation of RAD loci

Summaries of alignments and annotations to the *L. crocea* genome are provided in Table 4 and Table S2. Collectively, a total of 119 out of 126 RAD loci were matched to the *L. crocea* genome. A hit with a gene was obtained for 80.7% (96 loci) of these 119 RAD loci. Among them, 39 SNPs were located in exonic regions, 44 SNPs were located in intronic regions and 13 SNPs were located within 5 kb of coding sequences (CDS) regions. Based on a standard setting of gene count of 2, the enriched KEGG pathways are summarized in Table 4. The pathways containing the highest number of genes were AMP-activated protein kinase (AMPK) signaling pathway (five genes) and Endocytosis pathway (five genes). Other pathways of interest included glucagon signaling pathway (three genes), insulin signaling pathway (four genes) as well as regulation of actin cytoskeleton pathway (four genes). These candidate genes showed a broad functional categorization involved in multiple biological processes, such as growth and development, energy metabolism as well as cellular stress responses, etc.

**Table 4:** KEGG pathway analysis of the highly enriched categories.

Pathway term	ID	Gene count
AMPK signaling pathway	ko04152	5
Fc gamma R-mediated phagocytosis	ko04666	4
Insulin signaling pathway	ko04910	4
Endocytosis	ko04144	5
Shigellosis	ko05131	3
Focal adhesion	ko04510	4
Regulation of actin cytoskeleton	ko04810	4
Glucagon signaling pathway	ko04922	3
Insulin resistance	ko04931	3
Ubiquitin mediated proteolysis	ko04120	3
PI3K-Akt signaling pathway	ko04151	4
Proteoglycans in cancer	ko05205	3

## Discussion

### Genome-wide heterogeneous differentiation in *L. polyactis*

The significant finding in this study was the compelling support for shallow but significant genetic differentiation among overwintering populations of *L. polyactis* and suggesting the existence of hitherto undetected cryptic genetic structure within this fish species. It has remained uncertain whether *L. polyactis* are genetically structured in the Yellow Sea and the East China Sea (Liu, 1962; Xiao et al., 2009; Xu & Chen, 2009; Zhang et al., 2014). Our results demonstrated that the genetic differentiation is found to be driven in overwintering grounds. Namely, we found that the four overwintering populations formed three groups, including the Central Yellow Sea group (YA), the South Yellow Sea group (YB and YC) and the North East China Sea group (YD). And these results are consistent to the findings of Wang, Ma & You (1965) and Lin et al. (1964) based on fishery-dependent studies of *L. polyactis*. For both of these studies, 34°N and 32°N were regarded as geographic boundaries separating the Bohai Sea and Yellow Sea group, the South Yellow Sea group, and the East China Sea group of *L. polyactis*. In the present study, however, YC (31°02′30″N, 126°16′30″E) located in the south of Cheju island exhibited genetic similarity with the YB (32°59′49″N, 124°08′06″E) and these two sampling locations were observed to be clustered together as one group. Therefore, the southern geographical distribution boundary of South Yellow Sea group of *L. polyactis* might move southwards as compared to previous studies in 1960s. Bearing in mind that understanding population structure is critical for the conservation and management of natural resources (Lande, 1988; Petit, El Mousadik & Pons, 1998), overwintering stock of *L. polyactis* located to the north of 31°N might be incorporated as South Yellow Sea group fishery unit according to our study.

In winter and early spring, the Yellow Sea Warm Current (YSWC) originating from the northward Kuroshio and the Taiwan Warm Current (TWC) intrudes into the central Yellow Sea (Lin & Yang, 2011) (Fig. 1). A hydrological front emerges in the central Yellow Sea where the YSWC meets the cold water of the Yellow Sea Coastal Current (YSCC) (Fig. 1) (Chen, 2009; Lie, Cho & Lee, 2009; Lin & Yang, 2011). In this study, a clear genetic break was found between the East China Sea group and the other overwintering groups, which might be attributed to the isolation promoted by the hydrological front resulting from the intrusion of the high temperature and salinity YSWC. The front shows extreme contrasts between the cold fresh YSCC and warm saline YSWC, which may act as a barrier between East China Sea group and the Yellow Sea overwintering groups of *L. polyactis*. Oceanic fronts have been proven to represent common barriers to gene flow among the oceans and have a major influence on the population structure of marine fish (Galarza et al., 2009). In addition, a lower level of genetic divergence between the Central Yellow Sea group (YA) and the South Yellow Sea group (YB and YC) was also identified. Within South Yellow Sea group, both YB and YC populations were primarily located on the warm tongue of YSWC with a general trend of higher turbidity as vertical mixing plays an essential role in the formation of a warm water tongue of YSWC. In contrast, Central Yellow Sea group (YA) has much lower turbidity values (Table 1). Therefore, this observed divergence might be attributed to the environmental discontinuities of physical and biotic factors, which have an influence on genetic connectivity between the Central Yellow Sea group and the South Yellow Sea group of *L. polyactis*.

### Local adaptation to marine environment

Living in water, marine fish have intimate physiological contact with the environment at all times and variations in temperature, salinity and turbidity were widely reported to play important roles in shaping marine fish habitat (Nielsen et al., 2009a; Larmuseau et al., 2010; Guo, Li & Merilä, 2016). Therefore, three potential explanatory environmental variables (temperature, salinity and turbidity) were chosen to be tested for association with allele frequencies. Although the differences in salinity are to some extent slight between sites in our study, variable salinity was still kept for following association test. Because salinity is considered to be a key indicator of YSCWM which distributes in the lower layer in the Yellow Sea central trough with the salinity lower than 33 (Oh et al., 2013). Moreover, characteristics of the YSCWM can be shifted due to factors such as global climate change, which may play a critical role in determining the ecosystem change and thus affecting the overwintering distribution of marine fish in the Yellow Sea (Li et al., 2007). In spite of the generally low levels of genome wide genetic differentiation, several adaptive loci were also discovered, suggesting that local adaptation may be an important force in spite of the high levels of gene flow (Nielsen et al., 2009b). Our results stand in stark contrast to the findings from earlier studies that typically detected one or two loci associated with local adaptation (Wang et al., 2013; Liu et al., 2016). Genome scan approaches based on RAD-seq datasets have been used in previous studies to reveal adaptively important markers and genomic regions in marine fish with high levels of gene flow (Baltic Sea herring *Clupea harengus*, Guo, Li & Merilä, 2016; Pacific lamprey *Entosphenus tridentatus*, Hess et al., 2013; *Gadus morhua*, Bradbury et al., 2013), demonstrating its power for identifying signature of local adaptation in genomes of marine fish. In this study, testing for correlations between environmental parameters and allele frequencies identified several potentially adaptive loci which significantly correlated with local variables in temperature, salinity, and turbidity, suggesting these environmental factors may serve as selective forces driving genetic differentiation among overwintering populations.

AMPK system acts as a critical sensor of cellular energy status and can be activated by metabolic stresses such as deprivation for glucose as well as oxygen (Hardie & Sakamoto, 2006; Cantó et al., 2009). In general, activation of AMPK plays an important role in maintaining cellular energy stores and switching on catabolic pathways to generate ATP, such as upregulation of insulin secretion and decrease of glycolysis (Cantó et al., 2009). Indeed, three pathways involved in energy metabolism were also found in our study, including insulin signaling pathway, glucagon signaling pathway and insulin resistance pathway. The insulin signaling pathway is activated upon binding the hormone on its receptor and plays a key role in the regulation of storage and utilization of cellular glucose (Bevan, 2001). Gene functions of outlier RAD loci were found to be significantly enriched for the insulin signaling pathways in previous findings of European eel (*Anguilla anguilla*) and American Eel (*Anguilla rostrata*), which indicate that saccharide metabolism pathways may be among the key biological functions influenced by spatially varying selection in marine realm (Pujolar et al., 2015; Babin et al., 2017). In the present study, most of the candidate genes involved in these energy metabolism pathways were found to be significantly associated with turbidity. In winter, environmental turbidity conditions were found to be highly variable among overwintering grounds and the South Yellow Sea group (YB, YC) of *L. polyactis* have to experience continuously high turbidity episodes for months suffering from the vigorous vertical mixing by YSWC. Energy balance is a fundamental requirement of stress adaptation and tolerance. When an organism encounters environmental stresses, more energy is allocated for coping with stress-induced disturbance (Sokolova et al., 2012). Therefore, it is reasonable to speculate that large amounts of energy are necessary for surviving through overwintering season for the overwintering group of *L. polyactis* in South Yellow Sea. On the other hand, vertical mixing by the warm water tongue of YSWC greatly influences the turbidity in Southern part of Yellow Sea, which may bring nutrient-rich waters from the ocean bottom to the surface, supporting the growth of phytoplankton and seaweed which provides the energy base for zooplankton species and consumers higher in the food chain. Previous work has demonstrated that food availability can drive changes in ATP production and serves as a major driver of metabolic state (Dowd et al., 2013). Feeding ecology study on the diet composition of *L. polyactis* demonstrated that zooplankton species, especially crustaceans, were the most important prey groups and at the species level, euphausiids such as *Euphausia pacifica* were the most frequent prey (Xue et al., 2004). In overwintering seasons, YSWC greatly changes the zooplankton community structure in South Yellow Sea and *E. pacifica* was one of the top dominated species of zooplankton crustacean community, which may bring more food and nutrients supply for South Yellow Sea group of *L. polyactis* (Wang et al., 2003). Taken together, the overrepresentation of energy metabolism pathways here may play a role in the trade-offs inherent to occupying heterogeneous environmental regions affected by YSWC, which involves the regulation of energy reserve for growth.

Phagocytosis plays a critical role in host-defense mechanisms, which triggers the uptake and destruction of infectious pathogens and modulates phagosome formation by actin cytoskeleton rearrangements as well as membrane remodeling. Indeed, both regulation of actin cytoskeleton and endocytosis pathways were also found in the present study. Selective factors related to pathogen exposure were believed to be closely correlated with temperature or salinity (Shiah & Ducklow, 1994). As expected, most of the genes involved in defense responses were observed to be correlated with temperature and salinity (Table S2). Moreover, significant difference was discovered in the distribution characteristics of bacterioplankton among coasts of China and the distribution pattern was further observed to be associated with physico-chemical parameters such as temperature as well as salinity (Li et al., 2006; Ma et al., 2012). Therefore, genes involved in innate immune responses detected here may play a critical role in resistance to local pathogens for *L. polyactis*. In addition, the remaining genes not found in KEGG pathways also included genes associated with innate immune responses behavior, namely herc1 and ap3b1. Both genes were related to antigen processing and presentation associated with Major Histocompatibility Complex (MHC) (Rodgers & Cook, 2005). Teleost fish are considered to be the most primitive groups that possess the MHC genes which are crucial for the presentation of foreign-antigen peptides to the T-cell in the adaptive immune system (Manning & Nakanishi, 1996). Marine fish are free-living organisms in their aquatic environment and the innate response has been reported to play an essential role in combating a wide variety of pathogens and microorganisms (Uribe et al., 2011). In addition, it has been demonstrated in Atlantic salmon (*Salmo salar*) that the MHC polymorphism is maintained by pathogen-driven selection (Langefors et al., 2001). On the other hand, internal and external environmental factors such as temperature, salinity, water quality as well as other stress inducers can also greatly affect the immune responses of fish (Magnadottir, 2010). Therefore, genes involved in innate immune mechanisms could be important for the immune defenses to local pathogen exposure for *L. polyactis*.

## Conclusions

Comprehensive sampling combined with a genome-wide SNP markers in the present study revealed a shallow but significant genetic structure in the overwintering grounds of *L. polyactis* which has not been reported before. In the present study, we found that overwintering populations in the Yellow Sea and the North East China Sea formed three groups, including the Central Yellow Sea group (YA), the South Yellow Sea group (YB and YC) and the North East China Sea group (YD). A number of outlier loci were found to be significantly associated with various environmental factors despite the overall low levels of genome-wide genetic differentiation. We found evidence for adaptation to local temperature, salinity and turbidity in the overwintering grounds, which involved multiple biological processes including energy reserve for growth and innate immune defenses. Moreover, we denote that the patterns of genetic variation in high gene flow marine fish such as *L. polyactis* may be strongly influenced by ocean fronts as well as ocean currents, which are expected to increase the environmental heterogeneity on small spatial scales. It should be also noted that overwintering samples were collected from the same year in our study, and temporal stability in the genetic structure of *L. polyactis* will be further assessed in future studies. Nevertheless, our findings suggested that there might be three potential adaptive conservation units (CUs) in the overwintering grounds of *L. polyactis* and the adaptive markers detected here could be used to identify specific priorities for the future conservation of *L. polyactis* (Funk et al., 2012). Taken together, our results provide new insights into understanding of fine-scale population structure in *L. polyactis* and prove useful for elucidating potential population units as well as management implications for other marine migratory fish species.

##  Supplemental Information

10.7717/peerj.7242/supp-1 Figure S1 Bayesian Information Criterion (BIC) of DAPC analysis

10.7717/peerj.7242/supp-2 Figure S2 Plot of the first two principal components from PCA

10.7717/peerj.7242/supp-3 Figure S3 Cross-validation error of ADMIXTURE analysis

10.7717/peerj.7242/supp-4 Figure S4 The line diagram indicates the corresponding MEDMEDK, MEDMEAK, MAXMEDK and MAXMEAK statistics

10.7717/peerj.7242/supp-5 Table S1 Summary statistics of RAD data for each individual in this study

10.7717/peerj.7242/supp-6 Table S2 Summary of association testing of environmental variables with 21,620 SNP loci and annotation based on reference assembly of * L. crocea*

## Additional Information and Declarations

### Data Availability

The following information was supplied regarding data availability: Data is publicly accessible at GenBank under Bioproject accession number PRJNA533707 and Biosample accession numbers SAMN11489318–SAMN11489397.

## References

[ref-1] Alexander DH, Novembre J, Lange K (2009). Fast model-based estimation of ancestry in unrelated individuals. Genome Research.

[ref-2] Allendorf FW, Hohenlohe PA, Luikart G (2010). Genomics and the future of conservation genetics. Nature Reviews Genetics.

[ref-3] Ao J, Mu Y, Xiang LX, Fan D, Feng M, Zhang S, Shi Q, Zhu LY, Li T, Ding Y, Nie L, Li Q, Dong WR, Jiang L, Sun B, Zhang X, Li M, Zhang HQ, Xie S, Zhu Y, Jiang X, Wang X, Mu P, Chen W, Yue Z, Wang Z, Wang J, Shao JZ, Chen X (2015). Genome sequencing of the perciform fish *Larimichthys crocea* provides insights into molecular and genetic mechanisms of stress adaptation. PLOS Genetics.

[ref-4] Babin C, Gagnaire PA, Pavey SA, Bernatchez L (2017). RAD-seq reveals patterns of additive polygenic variation caused by spatially-varying selection in the American eel (*Anguilla rostrata*). Genome Biology & Evolution.

[ref-5] Baird NA, Etter PD, Atwood TS, Currey MC, Shiver AL, Lewis ZA, Selker EU, Cresko WA, Johnson EA (2008). Rapid SNP discovery and genetic mapping using sequenced RAD Markers. PLOS ONE.

[ref-6] Bevan P (2001). Insulin signaling. Journal of Cell Science.

[ref-7] Billington N, Hebert PDN (1990). Mitochondrial-DNA diversity in fishes and its implications for introductions. Canadian Journal of Fisheries and Aquatic Sciences.

[ref-8] Bradbury IR, Hubert S, Higgins B, Bowman S, Borza T, Paterson IG, Snelgrove PV, Morris CJ, Gregory RS, Hardie D, Hutchings JA, Ruzzante DE, Taggart CT, Bentzen P (2013). Genomic islands of divergence and their consequences for the resolution of spatial structure in an exploited marine fish. Evolutionary Applications.

[ref-9] Campbell D, Duchesne P, Bernatchez L (2003). AFLP utility for population assignment studies: analytical investigation and empirical comparison with microsatellites. Molecular Ecology.

[ref-10] Cano JM, Shikano T, Kuparinen A, Merilä J (2008). Genetic differentiation, effective population size and gene flow in marine fishes: implications for stock management. Journal of Integrative Field Biology.

[ref-11] Cantó C, Gerhart-Hines Z, Feige JN, Lagouge M, Noriega L, Milne JC, Elliott PJ, Puigserver P, Auwerx J (2009). AMPK regulates energy expenditure by modulating NAD+ metabolism and SIRT1 activity. Nature.

[ref-12] Catchen J, Hohenlohe PA, Bassham S, Amores A, Cresko WA (2013). Stacks: an analysis tool set for population genomics. Molecular Ecology.

[ref-13] Chen CTA (2009). Chemical and physical fronts in the Bohai, Yellow and East China seas. Journal of Marine Systems.

[ref-14] Chen JJ, Xu ZL, Chen XZ (2010). The spatial distribution pattern of fishing ground for small yellow croaker in China Seas. Journal of Fisheries of China.

[ref-15] Cheng JH, Lin LS, Ling JZ, Li JS, Ding FY (2004). Effects of summer close season and rational utilization of redlip croaker (*Larimichthys polyactis Bleeker*) resource in the East China Sea region. Journal of Fishery Sciences of China.

[ref-16] Conover DO, Clarke LM, Munich SB, Wagner GN (2006). Spatial and temporal scales of adaptive divergence in marine fishes and the implications for conservation. Journal of Fish Biology.

[ref-17] Coop G, Witonsky D, Di Rienzo A, Pritchard JK (2010). Using environmental correlations to identify loci underlying local adaptation. Genetics.

[ref-18] Corander J, Majander KK, Cheng L, Merilä J (2013). High degree of cryptic population differentiation in the Baltic Sea herring Clupea harengus. Molecular Ecology.

[ref-19] Danecek P, Auton A, Abecasis G, Albers CA, Banks E, DePristo MA, Handsaker RE, Lunter G, Marth GT, Sherry ST, McVean G, Durbin R (2011). The variant call format and VCFtools. Bioinformatics.

[ref-20] Davey JL, Blaxter MW (2010). RADSeq: next-generation population genetics. Briefings in Functional Genomics.

[ref-21] Ding FY, Lin LS, Li JS, Cheng JH (2007). Relationship between redlip croaker (*Larimichthys polyactis*) spawning stock distribution and water masses dynamics in northern East China Sea region. Journal of Natural Resources.

[ref-22] Dowd W, Felton CA, Heymann HM, Kost LE, Somero GN (2013). Food availability, more than body temperature, drives correlated shifts in ATP-generating and antioxidant enzyme capacities in a population of intertidal mussels (*Mytilus californianus*). Journal of Experimental Marine Biology and Ecology.

[ref-23] Etter PD, Bassham S, Hohenlohe PA, Johnson EA, Cresko WA (2011). SNP discovery and genotyping for evolutionary genetics using RAD sequencing. Methods in Molecular Biology.

[ref-24] Excoffier L, Lischer HEL (2010). Arlequin suite ver 3.5: a new series of programs to perform population genetics analyses under Linux and Windows. Molecular Ecology Resources.

[ref-25] Funk WC, Mckay JK, Hohenlohe PA, Allendorf FW (2012). Harnessing genomics for delineating conservation units. Trends in Ecology & Evolution.

[ref-26] Galarza JA, Carreras-Carbonell J, Macpherson E, Pascual M, Roques S, Turner GF, Rico C (2009). The influence of oceanographic fronts and early-life-history traits on connectivity among littoral fish species. Proceedings of the National Academy of Sciences of the United States of America.

[ref-27] Günther T, Coop G (2013). Robust identification of local adaptation from allele frequencies. Genetics.

[ref-28] Guo B, Li Z, Merilä J (2016). Population genomic evidence for adaptive differentiation in the Baltic Sea herring. Molecular Ecology.

[ref-29] Hardie DG, Sakamoto K (2006). AMPK: a key sensor of fuel and energy status in skeletal muscle. Physiology.

[ref-30] Hess JE, Campbell NR, Close DA, Docker MF, Narum SR (2013). Population genomics of Pacific lamprey: adaptive variation in a highly dispersive species. Molecular Ecology.

[ref-31] Hilborn R, Quinn TP, Schindler DE, Rogers DE (2003). Biocomplexity and fisheries sustainability. Proceedings of the National Academy of Sciences of the United States of America.

[ref-32] Huang DW, Sherman BT, Lempicki RA (2009a). Systematic and integrative analysis of large gene lists using DAVID Bioinformatics Resources. Nature Protocols.

[ref-33] Huang DW, Sherman BT, Lempicki RA (2009b). Bioinformatics enrichment tools: paths toward the comprehensive functional analysis of large gene lists. Nucleic Acids Research.

[ref-34] Ilut DC, Nydam ML, Hare MP (2014). Defining loci in restriction-based reduced representation genomic data from nonmodel species: sources of bias and diagnostics for optimal clustering. BioMed Research International.

[ref-35] Jarne P, Lagoda PJL (1996). Microsatellites, from molecules to populations and back. Trends in Ecology & Evolution.

[ref-36] Jin X (1996). Ecology and population dynamics of small yellow croaker (*Pseudosciaena Polyactis Bleeker*) in the Yellow Sea. Journal of Fishery Sciences of China.

[ref-37] Jin X (2004). Long-term changes in fish community structure in the Bohai Sea, China. Estuarine Coastal and Shelf Science.

[ref-38] Jombart T (2008). adegenet: a R package for the multivariate analysis of genetic markers. Bioinformatics.

[ref-39] Jombart T, Devillard S, Balloux F (2010). Discriminant analysis of principal components: a new method for the analysis of genetically structured populations. BMC Genetics.

[ref-40] Kim JK, Kim YH, Kim MJ, Park JY (2010). Genetic diversity, relationships and demographic history of the small yellow croaker, *Larimichthys polyactis* (Pisces: Sciaenidae) from Korea and China inferred from mitochondrial control region sequence data. Animal Cells and Systems.

[ref-41] Kim JK, Min GS, Yoon M, Kim Y, Choi JH, Oh TY, Ni Y (2012). Genetic structure of *Larimichthys polyactis* (Pisces: Sciaenidae) in the Yellow and East China Seas inferred from microsatellite and mitochondrial DNA analyses. Animal Cells and Systems.

[ref-42] Kopelman NM, Mayzel J, Jakobsson M, Rosenberg NA, Mayrose I (2015). CLUMPAK: A program for identifying clustering modes and packaging population structure inferences across K. Molecular Ecology Resources.

[ref-43] Lande R (1988). Genetics and demography in biological conservation. Science.

[ref-44] Langefors A, Lohm J, Grahn M, Andersen O, Von Schantz T (2001). Association between major histocompatibility complex class IIB alleles and resistance to *Aeromonas salmonicida* in Atlantic salmon. Proceedings. Biological Sciences.

[ref-45] Larmuseau MHD, Vancampenhout K, Raeymaekers JAM, Van Houdt JKJ, Volckaert FAM (2010). Differential modes of selection on the rhodopsin gene in coastal Baltic and North Sea populations of the sand goby, *Pomatoschistus minutus*. Molecular Ecology.

[ref-46] Le Moan A, Gagnaire PA, Bonhomme F (2016). Parallel genetic divergence among coastal-marine ecotype pairs of European anchovy explained by differential introgression after secondary contact. Molecular Ecology.

[ref-47] Li HB, Xiao T, Ding T, Lu RH (2006). The distribution of bacterioplankton in the Yellow Sea Cold Water Mass (YSCWM). Acta Ecologica Sinica.

[ref-48] Li JS, Lin LS, Cheng JH (2009). Distribution characteristic of small yellow croaker (*Larimichthys polyactis Bleeker*) and its relationship with bottom water temperature and salinity in the northern East China Sea in autumn. Journal of Fishery Sciences of China.

[ref-49] Li Y, Han Z, Song N, Gao TX (2013). New evidence to genetic analysis of small yellow croaker (*Larimichthys polyactis*) with continuous distribution in China. Biochemical Systematics and Ecology.

[ref-50] Li YL, Liu JX (2018). StructureSelector: a web-based software to select and visualize the optimal number of clusters using multiple methods. Molecular Ecology Resources.

[ref-51] Li YL, Xue DX, Zhang BD, Liu JX (2018). An optimized approach for local de novo assembly of overlapping paired-end RAD reads from multiple individuals. Royal Society Open Science.

[ref-52] Li Y, Zhao XY, Zhang T, Li XS, Wei H (2007). Wintering migration and distribution of anchovy in the Yellow Sea and its relation to physical environment. Marine Fisheries Research.

[ref-53] Lie HJ, Cho CH, Lee S (2009). Tongue-shaped frontal structure and warm water intrusion in the southern Yellow Sea in winter. Journal of Geophysical Research: Oceans.

[ref-54] Lin LS, Cheng JH (2004). An analysis of the current situation of fishery biology of small yellow croaker in the East China Sea. Journal of Ocean University of Qingdao.

[ref-55] Lin LS, Ying YP, Han ZQ, Xiao YS, Gao TX (2009). AFLP analysis on genetic diversity and population structure of small yellow croaker *Larimichthys polyactis*. African Journal of Biotechnology.

[ref-56] Lin XZ, Deng SM, Huang ZY, Wang QZ (1964). Study on fishery biology of *Larimichthys polyactis*. Selected Papers on Marine Fishery Resources.

[ref-57] Lin XP, Yang JY (2011). An asymmetric upwind flow, Yellow Sea Warm Current: 2. Arrested topographic waves in response to the northwesterly wind. Journal of Geophysical Research: Oceans.

[ref-58] Lin XP, Yang JY, Guo JS, Zhang ZX, Yin YQ, Song XZ, Zhang XH (2011). An asymmetric upwind flow, Yellow Sea Warm Current: 1. New observations in the western Yellow Sea. Journal of Geophysical Research Atmospheres.

[ref-59] Lischer HE, Excoffier L (2012). PGDSpider: an automated data conversion tool for connecting population genetics and genomics programs. Bioinformatics.

[ref-60] Liu BJ, Zhang BD, Xue DX, Gao TX, Liu JX (2016). Population structure and adaptive divergence in a high gene flow marine fish: the small yellow croaker (*Larimichthys polyactis*). PLOS ONE.

[ref-61] Liu X (1962). The research of small yellow croaker (*Larimichthys polyactis*) geographic race and gonad.

[ref-62] Luikart G, England PR, Tallmon D, Jordan S, Taberlet P (2003). The power and promise of population genomics: From genotyping to genome typing. Nature Reviews Genetics.

[ref-63] Ma C, Chen C, Jiang X, Li D, Liu M, Hao X, Qian Y (2012). Distribution characteristics of marine bacteria in the China seas. Medical Journal of Chinese People’s Liberation Army.

[ref-64] Magnadottir B (2010). Immunological control of fish diseases. Journal of Marine Biotechnology.

[ref-65] Manning MJ, Nakanishi T, Iwama GK, Nakanishi T (1996). Cellular defenses. Fish Fisiology. XV. The fish immune system.

[ref-66] Martin M (2011). Cutadapt removes adapter sequences from high-throughput sequencing reads. EMBnet Journal.

[ref-67] Meng ZN, Zhuang ZM, Jin XS, Tang QS, Su YQ (2003). Genetic diversity in small yellow croaker (*Pseudosciaena polyactis*) by RAPD analysis. Biodiversity Science.

[ref-68] Miller MR, Dunham JP, Amores A, Cresko WA, Johnson EA (2007). Rapid and cost-effective polymorphism identification and genotyping using restriction site associated DNA (RAD) markers. Genome Research.

[ref-69] Nichols KM, Kozfkay CC, Narum SR (2016). Genomic signatures among *Oncorhynchus nerka* ecotypes to inform conservation and management of endangered Sockeye Salmon. Evolutionary Applications.

[ref-70] Nielsen EE, Hemmer-Hansen J, Larsen PF, Bekkevold D (2009a). Population genomics of marine fishes: identifying adaptive variation in space and time. Molecular Ecology.

[ref-71] Nielsen EE, Hemmer-Hansen J, Poulsen NA, Loeschcke V, Moen T, Johansen T, Mittelholzer C, Taranger GL, Ogden R, Carvalho GR (2009b). Genomic signatures of local directional selection in a high gene flow marine organism; the Atlantic cod (*Gadus morhua*). BMC Evolutionary Biology.

[ref-72] Oh KH, Lee S, Song KM, Lie HJ, Kim YT (2013). The temporal and spatial variability of the Yellow Sea Cold Water Mass in the southeastern Yellow Sea, 2009-2011. Acta Oceanologica Sinica.

[ref-73] Pérez-Figueroa A, Garcia-Pereira MJ, Saura M, Rolan-Alvarez E, Caballero A (2010). Comparing three different methods to detect selective loci using dominant markers. Journal of Evolutionary Biology.

[ref-74] Petit RJ, El Mousadik A, Pons O (1998). Identifying populations for conservation on the basis of genetic markers. Conservation Biology.

[ref-75] Puechmaille SJ (2016). The program structure does not reliably recover the correct population structure when sampling is uneven: subsampling and new estimators alleviate the problem. Molecular Ecology Resources.

[ref-76] Pujolar JM, Jacobsen MW, Bekkevold D, Lobón-Cervià J, Jónsson B, Bernatchez L, Hansen MM (2015). Signatures of natural selection between life cycle stages separated by metamorphosis in European eel. BMC Genomics.

[ref-77] Pujolar JM, Jacobsen MW, Frydenberg J, Als TD, Larsen PF, Maes GE, Zane L, Jian JB, Cheng L, Hansen MM (2013). A resource of genome-wide single-nucleotide polymorphisms generated by RAD tag sequencing in the critically endangered European eel. Molecular Ecology Resources.

[ref-78] R Development Core Team (2015).

[ref-79] Rodgers JR, Cook RG (2005). MHC class Ib molecules bridge innate and acquired immunity. Nature Reviews Immunology.

[ref-80] Schindler DE, Hilborn R, Chasco B, Boatright CP, Quinn TP, Rogers LA, Webster MS (2010). Population diversity and the portfolio effect in an exploited species. Nature.

[ref-81] Schlitzer R (2019). Ocean Data View. https://odv.awi.de.

[ref-82] Shanks AL, Grantham BA, Carr MH (2003). Propagule dispersal distance and the size and spacing of marine reserves. Ecological Applications.

[ref-83] Shiah FK, Ducklow HW (1994). Temperature regulation of heterotrophic bacterioplankton abundance, production, and specific growth rate in Chesapeake Bay. Limnology and Oceanography.

[ref-84] Sokolova IM, Frederich M, Bagwe R, Lannig G, Sukhotin AA (2012). Energy homeostasis as an integrative tool for assessing limits of environmental stress tolerance in aquatic invertebrates. Marine Environmental Research.

[ref-85] Storz JF (2005). Using genome scans of DNA polymorphism to infer adaptive population divergence. Molecular Ecology.

[ref-86] Uribe C, Folch H, Enriquez R, Moran G (2011). Innate and adaptive immunity in teleost fish: a review. Veterinarni Medicina.

[ref-87] Wang L, Liu S, Zhuang Z, Guo L, Meng Z, Lin H (2013). Population genetic studies revealed local adaptation in a high gene-flow marine fish, the small yellow croaker (*Larimichthys polyactis*). PLOS ONE.

[ref-88] Wang R, Gao S, Wang K, Zuo T (2003). Zooplankton indication of the Yellow Sea Warm Current in winter. Journal of Fisheries of China.

[ref-89] Wang YG, Ma ZY, You HB (1965). Preliminary Study on Distribution and Migration Chracteristics of Small Yellow Croaker *Larimichthy polyactis* (Summary). Selected papers on marine fishery resources.

[ref-90] Wu R, Liu S, Zhuang Z, Su Y, Tang Q (2012). Population genetic structure and demographic history of small yellow croaker, *Larimichthys polyactis* (Bleeker, 1877), from coastal waters of China. African Journal of Biotechnology.

[ref-91] Xiao Y, Song N, Li J, Xiao Z, Gao T (2009). Genetic diversity in the mtDNA control region and population structure in the small yellow croaker *Larimichthys polyactis*. Environmental Biology of Fishes.

[ref-92] Xu ZL, Chen JJ (2009). Analysis on migratory routine of *Larimichthy polyactis*. Journal of Fishery Sciences of China.

[ref-93] Xu ZL, Chen JJ (2011). Population division of *Larimichthys polyactis* in China Sea. Chinese Journal of Applied Ecology.

[ref-94] Xue Y, Jin XS, Zhang B, Liang ZL (2004). Diet composition and seasonal variation in feeding habits of small yellow croaker in the central Yellow Sea. Journal of Fishery Sciences of China.

[ref-95] Yu F, Zhang ZX, Diao XY, Guo JS (2010). Observational evidence of the Yellow Sea warm current. Chinese Journal of Oceanology and Limnology.

[ref-96] Zhang C, Ye Z, Wan R, Ma Q, Li Z (2014). Investigating the population structure of small yellow croaker (*Larimichthys polyactis*) using internal and external features of otoliths. Fisheries Research.

