# Supplementary figures and images for: RAD genotyping reveals fine-scale population structure and provides evidence for adaptive divergence in a commercially important fish from the northwestern Pacific Ocean
Source: PeerJ. 2019 Jul 3;7:e7242. doi: 10.7717/peerj.7242 (PMC6612258; doi:10.7717/peerj.7242)

**Value of BIC  
versus number of clusters**

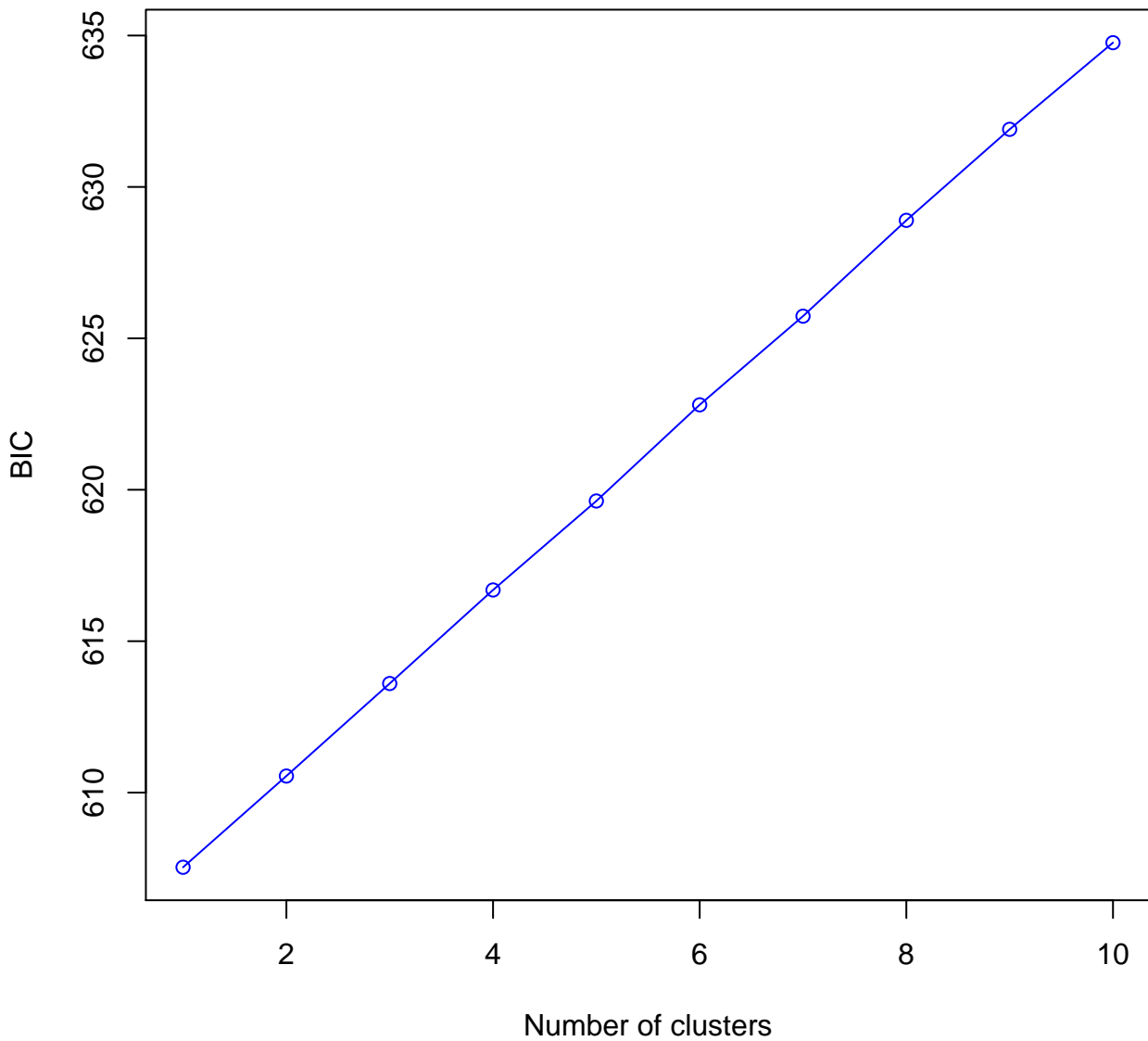

Supplement: Figure S1 [file peerj-07-7242-s001.pdf]

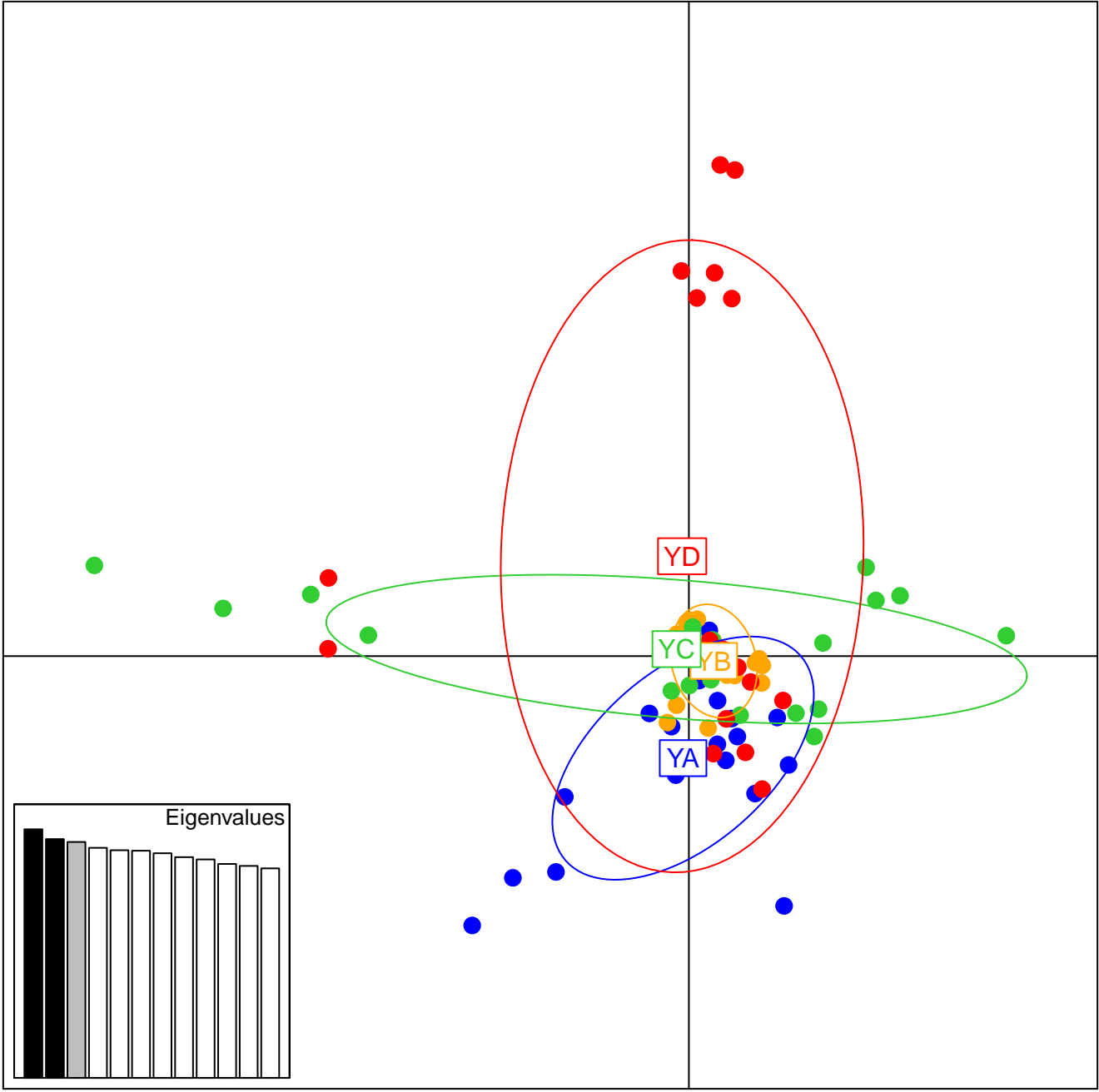

Supplement: Figure S2 [file peerj-07-7242-s002.pdf]

**CV error**

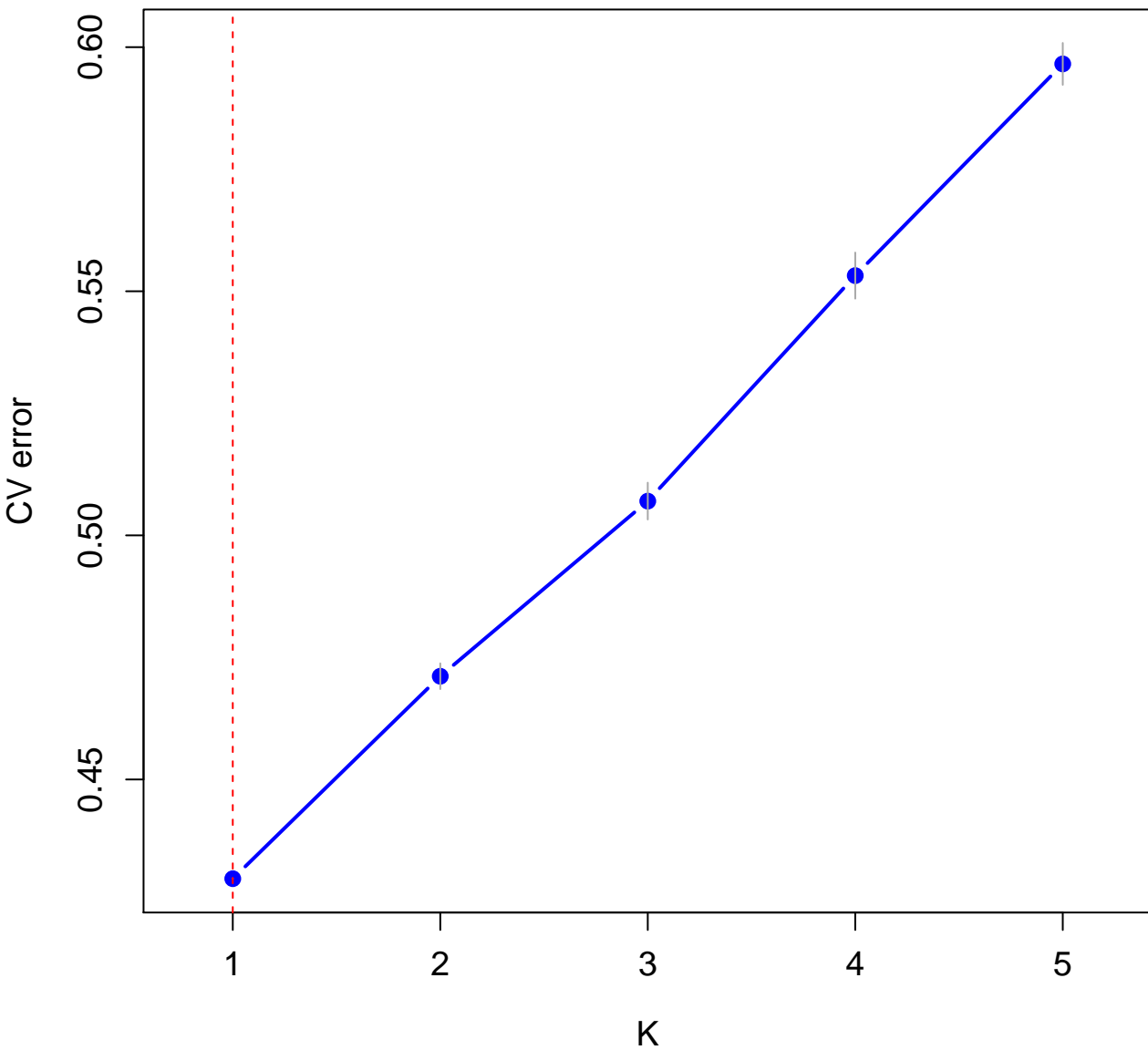

Supplement: Figure S3 [file peerj-07-7242-s003.pdf]

**MedMed K**

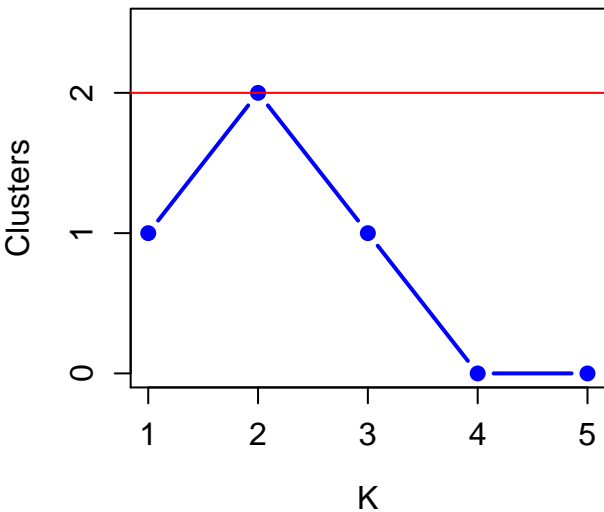

**MedMean K**

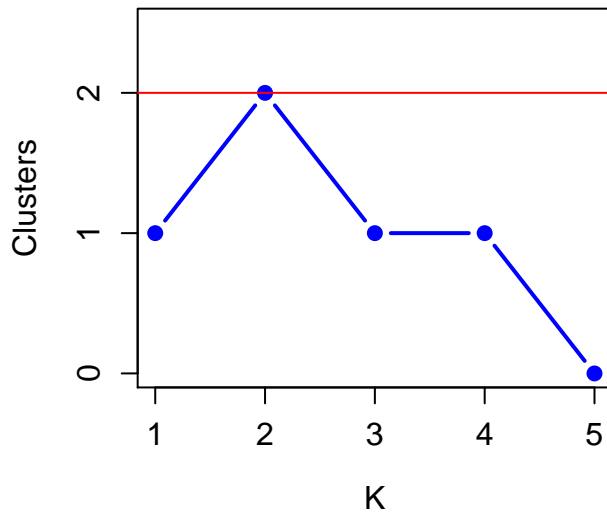

**MaxMed K**

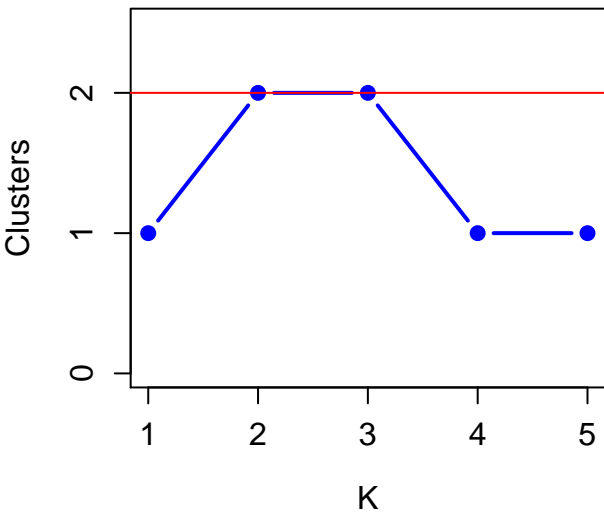

**MaxMean K**

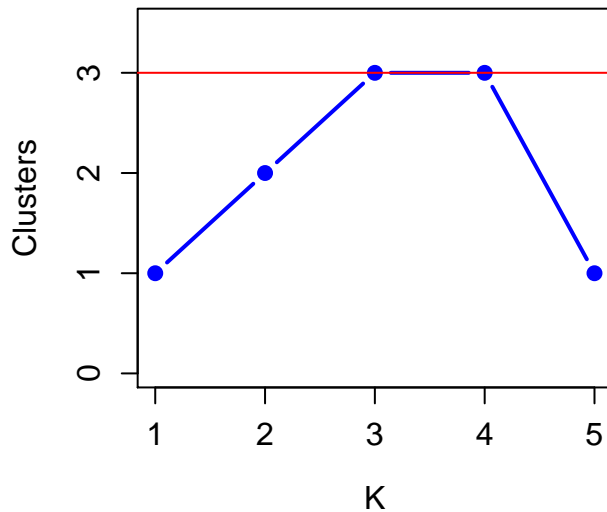

Supplement: Figure S4 [file peerj-07-7242-s004.pdf]
