# Supplementary material for: RAD genotyping reveals fine-scale population structure and provides evidence for adaptive divergence in a commercially important fish from the northwestern Pacific Ocean
Source: PeerJ. 2019 Jul 3;7:e7242. doi: 10.7717/peerj.7242 (PMC6612258; doi:10.7717/peerj.7242)
Supplement: Table S1 [file peerj-07-7242-s005.docx]

| No. | Sample | Raw read pair | Clean read pair | Clean reads | Clean data /G |
| --- | --- | --- | --- | --- | --- |
| 1 | XHY-YA-1 | 6,803,843 | 4,346,453 | 8,692,906 | 1.09 |
| 2 | XHY-YA-10 | 10,196,754 | 5,946,267 | 11,892,534 | 1.49 |
| 3 | XHY-YA-11 | 8,379,401 | 5,366,838 | 10,733,676 | 1.34 |
| 4 | XHY-YA-12 | 4,624,691 | 2,331,207 | 4,662,414 | 0.58 |
| 5 | XHY-YA-13 | 5,963,341 | 3,726,519 | 7,453,038 | 0.93 |
| 6 | XHY-YA-14 | 5,361,169 | 3,067,531 | 6,135,062 | 0.77 |
| 7 | XHY-YA-15 | 6,577,712 | 3,938,344 | 7,876,688 | 0.98 |
| 8 | XHY-YA-16 | 6,679,830 | 3,962,366 | 7,924,732 | 0.99 |
| 9 | XHY-YA-17 | 8,910,820 | 5,069,348 | 10,138,696 | 1.27 |
| 10 | XHY-YA-18 | 7,513,120 | 4,747,967 | 9,495,934 | 1.19 |
| 11 | XHY-YA-19 | 6,166,891 | 3,251,979 | 6,503,958 | 0.81 |
| 12 | XHY-YA-2 | 5,558,033 | 3,575,741 | 7,151,482 | 0.89 |
| 13 | XHY-YA-20 | 9,378,179 | 7,139,046 | 14,278,092 | 1.78 |
| 14 | XHY-YA-21 | 9,730,278 | 5,969,196 | 11,938,392 | 1.49 |
| 15 | XHY-YA-22 | 6,703,505 | 5,074,846 | 10,149,692 | 1.27 |
| 16 | XHY-YA-23 | 9,608,460 | 7,148,601 | 14,297,202 | 1.79 |
| 17 | XHY-YA-24 | 9,169,134 | 5,538,345 | 11,076,690 | 1.38 |
| 18 | XHY-YA-7 | 10,658,017 | 6,578,522 | 13,157,044 | 1.64 |
| 19 | XHY-YA-8 | 7,063,351 | 4,423,211 | 8,846,422 | 1.11 |
| 20 | XHY-YA-9 | 9,778,209 | 6,105,576 | 12,211,152 | 1.53 |
| 21 | XHY-YB-1 | 5,019,047 | 3,989,525 | 7,979,050 | 1 |
| 22 | XHY-YB-10 | 6,152,971 | 3,747,133 | 7,494,266 | 0.94 |
| 23 | XHY-YB-11 | 17469522 | 9,600,914 | 19,201,828 | 2.4 |
| 24 | XHY-YB-12 | 7,560,677 | 3,598,553 | 7,197,106 | 0.9 |
| 25 | XHY-YB-13 | 15,539,351 | 8,671,676 | 17,343,352 | 2.17 |
| 26 | XHY-YB-14 | 5,105,409 | 3,092,563 | 6,185,126 | 0.77 |
| 27 | XHY-YB-17 | 6,374,228 | 2,602,761 | 5,205,522 | 0.65 |
| 28 | XHY-YB-18 | 9,769,251 | 6,473,933 | 12,947,866 | 1.62 |
| 29 | XHY-YB-2 | 12,803,387 | 7,869,955 | 15,739,910 | 1.97 |
| 30 | XHY-YB-20 | 1,209,033 | 3,858,521 | 7,717,042 | 0.96 |
| 31 | XHY-YB-22 | 8,619,292 | 5,315,886 | 10,631,772 | 1.33 |
| 32 | XHY-YB-23 | 4,604,705 | 2,616,199 | 5,232,398 | 0.65 |
| 33 | XHY-YB-24 | 31,353,741 | 19,601,294 | 39,202,588 | 4.9 |
| 34 | XHY-YB-3 | 19,130,790 | 13,364,117 | 26,728,234 | 3.34 |
| 35 | XHY-YB-30 | 3,918,575 | 5,528,439 | 11,056,878 | 1.38 |
| 36 | XHY-YB-4 | 9,106,896 | 6,653,958 | 13,307,916 | 1.66 |
| 37 | XHY-YB-5 | 7,991,103 | 5,379,544 | 10,759,088 | 1.34 |
| 38 | XHY-YB-6 | 3,463,864 | 2,525,640 | 5,051,280 | 0.63 |
| 39 | XHY-YB-7 | 6,131,112 | 3,321,776 | 6,643,552 | 0.83 |
| 40 | XHY-YB-8 | 6,260,795 | 4,141,125 | 8,282,250 | 1.04 |
| 41 | XHY-YC-1 | 8,902,392 | 5,127,632 | 10,255,264 | 1.28 |
| 42 | XHY-YC-10 | 3,466,663 | 3,747,133 | 7,494,266 | 0.94 |
| 43 | XHY-YC-11 | 11,474,897 | 7,155,013 | 14,310,026 | 1.79 |
| 44 | XHY-YC-12 | 11,888,465 | 8,342,444 | 16,684,888 | 2.09 |
| 45 | XHY-YC-13 | 8,443,076 | 5,944,420 | 11,888,840 | 1.49 |
| 46 | XHY-YC-16 | 5,816,068 | 4,225,247 | 8,450,494 | 1.06 |
| 47 | XHY-YC-17 | 8,813,875 | 6,111,537 | 12,223,074 | 1.53 |
| 48 | XHY-YC-18 | 6,584,627 | 3,874,836 | 7,749,672 | 0.97 |
| 49 | XHY-YC-2 | 8,602,520 | 4,982,363 | 9,964,726 | 1.25 |
| 50 | XHY-YC-20 | 8,500,513 | 5,322,293 | 10,644,586 | 1.33 |
| 51 | XHY-YC-22 | 3,512,442 | 2,288,245 | 4,576,490 | 0.57 |
| 52 | XHY-YC-24 | 8,865,411 | 5,640,930 | 11,281,860 | 1.41 |
| 53 | XHY-YC-25 | 10,843,360 | 7,759,716 | 15,519,432 | 1.94 |
| 54 | XHY-YC-27 | 12,682,896 | 8,518,545 | 17,037,090 | 2.13 |
| 55 | XHY-YC-3 | 17,739,383 | 16,592,752 | 33,185,504 | 4.15 |
| 56 | XHY-YC-4 | 8,992,408 | 5,537,767 | 11,075,534 | 1.38 |
| 57 | XHY-YC-5 | 9,620,544 | 5,856,104 | 11,712,208 | 1.46 |
| 58 | XHY-YC-6 | 2,358,006 | 2,200,679 | 4,401,358 | 0.55 |
| 59 | XHY-YC-7 | 5,850,027 | 3,777,373 | 7,554,746 | 0.94 |
| 60 | XHY-YC-8 | 13,702,158 | 13,744,064 | 27,488,128 | 3.44 |
| 61 | XHY-YD-1 | 5,968,332 | 3,825,959 | 7,651,918 | 0.96 |
| 62 | XHY-YD-12 | 7,210,325 | 4,446,261 | 8,892,522 | 1.11 |
| 63 | XHY-YD-13 | 5,617,623 | 3,464,310 | 6,928,620 | 0.87 |
| 64 | XHY-YD-14 | 8,532,023 | 5,301,405 | 10,602,810 | 1.33 |
| 65 | XHY-YD-15 | 7,011,064 | 4,217,078 | 8,434,156 | 1.05 |
| 66 | XHY-YD-16 | 7,753,640 | 4,965,994 | 9,931,988 | 1.24 |
| 67 | XHY-YD-17 | 7,969,725 | 4,832,692 | 9,665,384 | 1.21 |
| 68 | XHY-YD-18 | 7,957,537 | 4,958,204 | 9,916,408 | 1.24 |
| 69 | XHY-YD-19 | 8,494,230 | 5,261,060 | 10,522,120 | 1.32 |
| 70 | XHY-YD-2 | 4,095,084 | 2,656,818 | 5,313,636 | 0.66 |
| 71 | XHY-YD-20 | 9,487,177 | 5,870,795 | 11,741,590 | 1.47 |
| 72 | XHY-YD-21 | 6,372,075 | 3,631,041 | 7,262,082 | 0.91 |
| 73 | XHY-YD-22 | 9,434,436 | 5,646,358 | 11,292,716 | 1.41 |
| 74 | XHY-YD-23 | 10,788,681 | 6,656,088 | 13,312,176 | 1.66 |
| 75 | XHY-YD-24 | 4,964,450 | 2,902,901 | 5,805,802 | 0.73 |
| 76 | XHY-YD-3 | 7,982,001 | 4,884,405 | 9,768,810 | 1.22 |
| 77 | XHY-YD-4 | 8,461,227 | 5,336,944 | 10,673,888 | 1.33 |
| 78 | XHY-YD-5 | 10,813,230 | 6,318,187 | 12,636,374 | 1.58 |
| 79 | XHY-YD-6 | 4,738,280 | 3,019,518 | 6,039,036 | 0.75 |
| 80 | XHY-YD-9 | 7,885,782 | 4,813,995 | 9,627,990 | 1.2 |
