# Supplementary material for: RAD genotyping reveals fine-scale population structure and provides evidence for adaptive divergence in a commercially important fish from the northwestern Pacific Ocean
Source: PeerJ. 2019 Jul 3;7:e7242. doi: 10.7717/peerj.7242 (PMC6612258; doi:10.7717/peerj.7242)
Supplement: Table S2 [file peerj-07-7242-s006.docx]

|  | | | | | | | | |
| --- | --- | --- | --- | --- | --- | --- | --- | --- |
| Contig | NCBI Acession | Gene ID | Gene symbol | Gene description | KEGG pathway | TEMP | SAL | TUB |
| 8039 | NW_017609806.1 | 104933254 | ints10 | integrator complex subunit 10 | - | * | * | - |
| 8345 | NW_017608489.1 | 104931535 | LOC104931535 | adhesion G protein-coupled receptor L2-like | - | * | - | - |
| 71108 | NW_017607965.1 | 104928354 | LOC104928354 | LIM domain and actin-binding protein 1-like | - | * | - | - |
| 144086 | NW_017607960.1 | 104926617 | ap3b1 | adaptor related protein complex 3 subunit beta 1 | - | * | * | - |
| 19712 | NW_017608489.1 | 104918299 | znf521 | zinc finger protein 521 | - | * | * | - |
| 147216 | NW_017609049.1 | 104927344 | fes | FES proto-oncogene, tyrosine kinase | - | * | * | - |
| 54002 | NW_017608764.1 | 104939391 | LOC104939391 | thyroid hormone receptor-associated protein 3-like | - | * | * | - |
| 74583 | NW_017608919.1 | 104920712 | cog7 | component of oligomeric golgi complex 7 | - | * | - | - |
| 105113 | NW_017608616.1 | 104922104 | wasf2 | WAS protein family member 2 | Fc gamma R-mediated phagocytosis/ Regulation of actin cytoskeleton | * | * | - |
| 31111 | NW_017609869.1 | 104931627 | LOC104931627 | synaptic vesicle glycoprotein 2A | - | * | - | - |
| 181876 | NW_017607886.1 | 104924686 | LOC104924686 | calmodulin-binding transcription activator 1-like | - | * | - | - |
| 73915 | NW_017608811.1 | 104934438 | ppp4r4 | protein phosphatase 4 regulatory subunit 4 | - | * | * | - |
| 152236 | NW_017608229.1 | 104918566 | camsap3 | calmodulin regulated spectrin associated protein family member 3 | - | * | * | - |
| 164393 | NW_017608620.1 | 104924419 | jade2 | jade family PHD finger 2 | - | * | - | - |
| 89796 | NW_017609900.1 | 104934976 | gpa33 | glycoprotein A33 | - | * | - | - |
| 29411 | NW_017609507.1 | 104935458 | LOC104935458 | septin-4 | - | * | - | - |
| 91839 | NW_017609030.1 | 104927653 | LOC104927653 | P2Y purinoceptor 13 | - | * | - | - |
| 111467 | NW_017609029.1 | 104918482 | LOC104918482 | leucine-rich repeat and immunoglobulin-like domain-containing nogo receptor-interacting protein 3 | - | * | - | - |
| 80663 | NW_017608852.1 | 104935760 | herc1 | HECT and RLD domain containing E3 ubiquitin protein ligase family member 1 | Ubiquitin mediated proteolysis | * | - | - |
| 189443 | NW_017608645.1 | 104926550 | LOC104926550 | sodium/potassium-transporting ATPase subunit alpha-2-like | - | * | - | - |
| 86387 | NW_017608756.1 | 104934221 | LOC104934221 | solute carrier family 22 member 2-like | - | * | * | - |
| 108086 | NW_017608983.1 | 109140885 | LOC109140885 | homeodomain-interacting protein kinase 1-like | - | * | - | - |
| 190952 | NW_017609028.1 | 104918624 | itga9 | integrin subunit alpha 9 | Focal adhesion/ Regulation of actin cytoskeleton/ PI3K-Akt signaling pathway | * | - | - |
| 125519 | NW_017608252.1 | 104924338 | sorl1 | sortilin related receptor 1 | - | * | - | - |
| 34375 | NW_017608364.1 |  |  |  | - | * | - | - |
| 161321 | NW_017608793.1 | 104920408 | krt222 | keratin 222 | - | * | * | - |
| 20487 | NW_017608878.1 | 104939540 | lmod1 | leiomodin 1 | - | * | - | - |
| 118855 | NW_017608812.1 | 104931864 | mrpl37 | mitochondrial ribosomal protein L37 | - | * | - | - |
| 55563 | NW_017608755.1 | 104932033 | ncam2 | neural cell adhesion molecule 2 | - | * | - | - |
| 122584 | NW_017609445.1 | 104928205 | LOC104928205 | protein furry homolog | - | * | - | - |
| 96569 | NW_017608148.1 | 104927106 | LOC104927106 | tumor suppressor candidate 3 | - | * | - | - |
| 117083 | NW_017608088.1 | 104930146 | LOC104930146 | thyrotropin-releasing hormone receptor-like | - | * | - | - |
| 58331 | NW_017609651.1 | 104929775 | slc8a3 | solute carrier family 8 member A3 | - | * | - | - |
| 191829 | NW_017608596.1 | 104936967 | LOC104936967 | ataxin-7-like protein 2/atxn7l2 | - | * | - | - |
| 34964 | NW_017609429.1 | 104921500 | ska3 | spindle and kinetochore associated complex subunit 3 | - | * | - | - |
| 131241 | NW_017608968.1 | 104923860 | LOC104923860 | zinc finger protein 64 homolog, isoforms 1 and 2 | - | * | * | - |
| 10520 | NW_017609596.1 | 104921830 | LOC104921830 | actin-related protein 2/3 complex subunit 5-like | Endocytosis/ Fc gamma R-mediated phagocytosis/ Regulation of actin cytoskeleton | * | - | - |
| 36240 | NW_017609241.1 | 104934955 | gle1 | GLE1, RNA export mediator | - | * | - | - |
| 128525 | NW_017609378.1 | 104918159 | mmp16 | matrix metallopeptidase 16 | - | * | - | - |
| 196683 | NW_017611226.1 | 104931311 | tbc1d9 | TBC1 domain family member 9 | - | * | - | - |
| 164463 | NW_017608125.1 | 104923745 | net1 | neuroepithelial cell transforming 1 | - | * | - | - |
| 148445 | NW_017609378.1 | 104924700 | LOC104924700 | double-stranded RNA-specific editase B2 | - | * | - | - |
| 98042 | NW_017608201.1 | 104928046 | dcc | DCC netrin 1 receptor | - | * | - | - |
| 159794 | NW_017613856.1 | 104919397 | clstn2 | calsyntenin 2 | - | * | * | - |
| 31373 | NW_017608610.1 | 104930407 | diaph1 | diaphanous related formin 1 | Focal adhesion/ Regulation of actin cytoskeleton | - | * | - |
| 121612 | NW_017609519.1 | 104936919 | LOC104936919 | barrier-to-autointegration factor | - | - | * | - |
| 36802 | NW_017609447.1 | 104929502 | LOC104929502 | transcription factor IIIB 90 kDa subunit-like | - | - | * | - |
| 154853 | NW_017607935.1 | 104925875 | dnah7 | dynein axonemal heavy chain 7 | - | - | * | - |
| 179384 | NW_017609274.1 | 109141694 | LOC109141694 | NADH dehydrogenase [ubiquinone] iron-sulfur protein 4, mitochondrial-like | - | - | * | - |
| 118232 | NW_017608646.1 | 104918410 | znf236 | zinc finger protein 236 | - | - | * | - |
| 84981 | NW_017609670.1 | 109142941 | LOC109142941 | cadherin-13-like | - | - | * | - |
| 40937 | NW_017608305.1 | 104934171 | LOC104934171 | KH domain-containing, RNA-binding, signal transduction-associated protein 1-like | - | - | * | - |
| 137511 | NW_017609827.1 | 104931428 | LOC104931428 | epidermal growth factor receptor substrate 15-like 1 | Endocytosis | - | * | - |
| 66734 | NW_017607886.1 | 109137320 | ppp1r3f | protein phosphatase 1 regulatory subunit 3F | Insulin signaling pathway | - | * | - |
| 183602 | NW_017608098.1 | 109138114 | LOC109138114 | uncharacterized LOC109138114 | - | - | * | - |
| 105501 | NW_017609681.1 | 104920470 | LOC104920470 | carnitine O-palmitoyltransferase 1, liver isoform | AMPK signaling pathway/ Glucagon signaling pathway/  Insulin resistance | - | * | - |
| 60465 | NW_017608170.1 | 104921896 | LOC104921896 | sorbin and SH3 domain-containing protein 1-like | - | - | * | - |
| 108321 | NW_017609723.1 | 104936307 | cptp | ceramide-1-phosphate transfer protein | - | - | * | - |
| 113131 | NW_017609176.1 | 104925300 | pou6f2 | POU class 6 homeobox 2 | - | - | * | - |
| 91882 | NW_017608489.1 | 104918287 | LOC104918287 | pre-B-cell leukemia transcription factor 1 | - | - | * | - |
| 159636 | NW_017608489.1 | 104929640 | LOC104929640 | nuclear receptor ROR-beta-like | - | - | * | - |
| 116080 | NW_017608491.1 | 104927820 | insc | INSC, spindle orientation adaptor protein | - | - | * | - |
| 141455 | NW_017609652.1 | 104934512 | abcg4 | ATP binding cassette subfamily G member 4 | - | - | * | - |
| 11534 | NW_017609289.1 | 104932418 | LOC104932418 | USP6 N-terminal-like protein | - | - | * | - |
| 81707 | NW_017609517.1 | 104925871 | nabp1 | nucleic acid binding protein 1 | - | - | - | * |
| 32042 | NW_017607908.1 | 104922233 | LOC104922233 | neuron-specific protein family member 1-like | - | - | - | * |
| 68656 | NW_017607933.1 | 104930479 | LOC104930479 | serine/threonine-protein kinase NIM1-like | - | - | - | * |
| 127580 | NW_017607909.1 | 104917931 | LOC104917931 | CUGBP Elav-like family member 2 | - | - | - | * |
| 175380 | NW_017608691.1 | 104933139 | LOC104933139 | ral GTPase-activating protein subunit beta-like | - | - | - | * |
| 135085 | NW_017609237.1 | 104934742 | LOC104934742 | solute carrier family 12 member 2-like | - | - | - | * |
| 56936 | NW_017609517.1 | 109142558 | LOC109142558 | protein EFR3 homolog A-like | - | - | - | * |
| 30054 | NW_017607967.1 | 104928803 | LOC104928803 | protein piccolo-like | - | - | - | * |
| 159272 | NW_017609651.1 | 104938237 | pygl | glycogen phosphorylase L | Insulin signaling pathway/ Glucagon signaling pathway/ Insulin resistance | - | - | * |
| 178508 | NW_017608922.1 | 104939054 | LOC104939054 | bone morphogenetic protein 7-like | - | - | - | * |
| 34675 | NW_017608766.1 | 109140134 | LOC109140134 | F-box/LRR-repeat protein 17-like | - | - | - | * |
| 49894 | NW_017609716.1 | 104927029 | ankrd27 | ankyrin repeat domain 27 | - | - | - | * |
| 41176 | NW_017609703.1 | 104933422 | iglon5 | IgLON family member 5 | - | - | - | * |
| 61375 | NW_017608616.1 | 104922065 | cunh18orf21 | chromosome unknown C18orf21 homolog | - | - | - | * |
| 126532 | NW_017609670.1 | 109142943 | LOC109142943 | uncharacterized LOC109142943 | - | - | - | * |
| 67052 | NW_017609304.1 | 104919896 | LOC104919896 | integrin alpha-5-like | - | - | - | * |
| 102969 | NW_017613860.1 | 104931579 | ubr5 | ubiquitin protein ligase E3 component n-recognin 5 | Ubiquitin mediated proteolysis | - | - | * |
| 57010 | NW_017608859.1 | 104937345 | retreg1 | reticulophagy regulator 1 | - | - | - | * |
| 165034 | NW_017608859.1 | 104930077 | LOC104930077 | receptor-type tyrosine-protein phosphatase N2-like | - | - | - | * |
| 69202 | NW_017607880.1 | 104922389 | LOC104922389 | complexin-1-like | - | - | - | * |
| 66009 | NW_017609274.1 | 109141690 | LOC109141690 | gamma-aminobutyric acid receptor subunit beta-3-like | - | - | - | * |
| 52835 | NW_017608696.1 | 104939201 | LOC104939201 | ELAV-like protein 1 | AMPK signaling pathway | - | - | * |
| 75996 | NW_017609304.1 | 104921544 | rptor | egulatory associated protein of MTOR complex 1 | AMPK signaling pathway/ Insulin signaling pathway/ PI3K-Akt signaling pathway | - | - | * |
| 117824 | NW_017608121.1 | 104936766 | tmeff1 | transmembrane protein with EGF like and two follistatin like domains 1 | - | - | - | * |
| 67458 | NW_017607998.1 | 104921706 | LOC104921706 | insulin-like growth factor 1 receptor | AMPK signaling pathway/ Endocytosis/ Focal adhesion/ PI3K-Akt signaling pathway/  Proteoglycans in cancer | - | - | * |
| 37019 | NW_017608302.1 | 104918516 | LOC104918516 | RAC-gamma serine/threonine-protein kinase-like | AMPK signaling pathway/ Fc gamma R-mediated phagocytosis/ Insulin signaling pathway/ Focal adhesion/ Glucagon signaling pathway/ Insulin resistance/ PI3K-Akt signaling pathway/  Proteoglycans in cancer | - | - | * |
| 174646 | NW_017609842.1 | 104932493 | ube4b | ubiquitination factor E4B | Ubiquitin mediated proteolysis | - | - | * |
| 175414 | NW_017609269.1 | 104925965 | tenm4 | teneurin transmembrane protein 4 | - | - | - | * |
| 189297 | NW_017609176.1 | 104929598 | asap1 | ArfGAP with SH3 domain, ankyrin repeat and PH domain 1 | Endocytosis/ Fc gamma R-mediated phagocytosis | - | - | * |
| 106341 | NW_017609732.1 | 104937733 | clvs2 | clavesin 2 | - | - | - | * |
| 173280 | NW_017609123.1 | 104920781 | ftcd | formimidoyltransferase cyclodeaminase | - | - | - | * |
| 125054 | NW_017609606.1 | 104929362 | erbb4 | erb-b2 receptor tyrosine kinase 4 | Endocytosis/  Proteoglycans in cancer | - | - | * |
| 148817 | NW_017609824.1 | 104931496 | ralyl | RALY RNA binding protein like | - | - | - | * |
|  |  |  |  |  |  |  |  |  |
